# Longitudinal Trajectories of Quality of Life Among People With Mild-to-Moderate Dementia: A Latent Growth Model Approach With IDEAL Cohort Study Data

**DOI:** 10.1093/geronb/gbac022

**Published:** 2022-02-03

**Authors:** Linda Clare, Laura D Gamble, Anthony Martyr, Serena Sabatini, Sharon M Nelis, Catherine Quinn, Claire Pentecost, Christina Victor, Roy W Jones, Ian R Jones, Martin Knapp, Rachael Litherland, Robin G Morris, Jennifer M Rusted, Jeanette M Thom, Rachel Collins, Catherine Henderson, Fiona E Matthews

**Affiliations:** Centre for Research in Ageing and Cognitive Health, University of Exeter Medical School, Exeter, UK; NIHR Applied Research Collaboration South-West Peninsula, Exeter, UK; Population Health Sciences Institute, Newcastle University, Newcastle upon Tyne, UK; Centre for Research in Ageing and Cognitive Health, University of Exeter Medical School, Exeter, UK; Centre for Research in Ageing and Cognitive Health, University of Exeter Medical School, Exeter, UK; Centre for Research in Ageing and Cognitive Health, University of Exeter Medical School, Exeter, UK; Centre for Applied Dementia Studies, Bradford University, Bradford, UK; Wolfson Centre for Applied Health Research, Bradford, UK; Centre for Research in Ageing and Cognitive Health, University of Exeter Medical School, Exeter, UK; College of Health, Medicine and Life Sciences, Brunel University London, London, UK; Research Institute for the Care of Older People (RICE), Bath, UK; Wales Institute for Social and Economic Research, Data and Methods, Cardiff University, Cardiff, UK; Care Policy and Evaluation Centre, London School of Economics and Political Science, London, UK; Innovations in Dementia CIC, Exeter, UK; Institute of Psychiatry, Psychology and Neuroscience, King’s College London, London, UK; School of Psychology, University of Sussex, Brighton, UK; School of Health Sciences, University of New South Wales, Sydney, Australia; Centre for Research in Ageing and Cognitive Health, University of Exeter Medical School, Exeter, UK; Care Policy and Evaluation Centre, London School of Economics and Political Science, London, UK; Population Health Sciences Institute, Newcastle University, Newcastle upon Tyne, UK

**Keywords:** Alzheimer’s, Caregivers, Longitudinal

## Abstract

**Objectives:**

We aimed to examine change over time in self-rated quality of life (QoL) in people with mild-to-moderate dementia and identify subgroups with distinct QoL trajectories.

**Methods:**

We used data from people with mild-to-moderate dementia followed up at 12 and 24 months in the Improving the experience of Dementia and Enhancing Active Life (IDEAL) cohort study (baseline *n* = 1,537). A latent growth model approach examined mean change over time in QoL, assessed with the QoL-AD scale, and investigated associations of baseline demographic, cognitive, and psychological covariates with the intercept and slope of QoL. We employed growth mixture modeling to identify multiple growth trajectories.

**Results:**

Overall mean QoL scores were stable and no associations with change over time were observed. Four classes of QoL trajectories were identified: 2 with higher baseline QoL scores, labeled Stable (74.9%) and Declining (7.6%), and 2 with lower baseline QoL scores, labeled Stable Lower (13.7%) and Improving (3.8%). The Declining class had higher baseline levels of depression and loneliness, and lower levels of self-esteem and optimism, than the Stable class. The Stable Lower class was characterized by disadvantage related to social structure, poor physical health, functional disability, and low psychological well-being. The Improving class was similar to the Stable Lower class but had lower cognitive test scores.

**Discussion:**

Understanding individual trajectories can contribute to personalized care planning. Efforts to prevent decline in perceived QoL should primarily target psychological well-being. Efforts to improve QoL for those with poorer QoL should additionally address functional impairment, isolation, and disadvantage related to social structure.

Of the 50 million people living with dementia worldwide, most live in the community. Enabling them to experience a good quality of life (QoL) and “live well” (Institute of Medicine, 2012, p. 32) is important. QoL reflects people’s “perception of their position in life in the context of the culture and value systems in which they live and in relation to their goals, expectations, standards and concerns” (WHOQOL Group, 1993, p. 153).

QoL is subject to multiple influences; self-ratings of QoL by people with dementia have weak cross-sectional associations with numerous factors (Martyr et al., 2018). In the Improving the experience of Dementia and Enhancing Active Life (IDEAL) study of people living with mild-to-moderate dementia and their informal caregivers in Britain (Clare, Nelis, et al., 2014), we sought to provide a more comprehensive picture of these associations, drawing on Lawton’s formulation of QoL (Lawton, 1994). Modeling based on cross-sectional data (Clare et al., 2019) demonstrated the independent association of five life domains with QoL, measured using the Quality of Life in Alzheimer’s Disease (QoL-AD) scale (Logsdon et al., 2000). When all domains were modeled together, only the psychological domain remained independently associated with QoL.

Understanding these patterns of cross-sectional associations is valuable. However, a longitudinal perspective is essential for understanding how experiences change over time and what factors drive any such change. Current evidence about baseline predictors of later QoL in people with dementia is limited (Martyr et al., 2018). There are several reasons for this. Existing studies of longitudinal change in self-rated QoL have significant limitations; many report only a single follow-up, apply only basic statistical methods, and include relatively small samples. Second, most studies assessing QoL longitudinally, and employing measures based on a broad conceptualization of QoL such as the QoL-AD (Logsdon et al., 2000) or Dementia Quality of Life (DEMQoL) scale (Smith et al., 2007), have compared mean scores for the whole sample across time points and reported no overall change (Andrieu et al., 2016; Bosboom & Almeida, 2016; Clare et al., 2012; Clare, Woods, et al., 2014; Conde-Sala et al., 2016; Dourado et al., 2016; Hongisto et al., 2015; Livingston et al., 2008; O’Shea et al., 2020; Sousa et al., 2018; Trigg et al., 2015). If scores are stable this leaves little scope to identify predictors of change. There is only one exception to the finding of overall stability; Kisvetrová et al. (2020) reported a mean decline of 1.98 points on the QoL-AD at 24 months. This could be attributable to sample characteristics (e.g., participants had been diagnosed in the last 12 months, and there was a nonsignificant increase in depression scores at 24 months) or cultural circumstances (participants were resident in the Czech Republic). Whatever the reason, this finding indicates that stability in group-level mean scores cannot necessarily be assumed. Third, few studies have looked beyond group-level findings. Three studies, all reporting no overall change, have investigated whether observed stability in mean scores at group level masks individual variation. Two examined change from baseline in responses to either a single QoL-AD item (Livingston et al., 2008) or total DEMQoL score (Trigg et al., 2015) at 18 month follow-up. Both noted variation in individual scores, but considered a one-step shift in response option or a one-point change in total score, respectively, a sufficient indicator of change. Clare, Woods, et al. (2014) calculated a reliable change index for QoL-AD scores in the Memory Impairment and Dementia Awareness study (MIDAS) sample (*n* = 51), indicating that only changes in total score of 6 points or more were reliable. Using this criterion, at 20-month follow-up 76% had a stable trajectory, 12% improved, and 12% declined. Further work with a larger sample might identify groups with different trajectories more reliably, and if so, indicate which factors predict subsequent change in QoL and suggest ways of preventing decline.

In summary, available evidence suggests it may be informative to look beyond group-level mean scores and explore within-sample variation in QoL scores over time, while addressing the methodological limitations of previous research. In this study, using data from the cohort of people with mild-to-moderate dementia followed up at 12 and 24 months in IDEAL, employing robust modeling methods and applying a reliable change index, we aimed first to identify the extent to which self-rated QoL changes over time for the whole cohort and to clarify whether groups with different QoL trajectories could be reliably identified. If so, our objective was to profile these groups clustered according to QoL trajectories, and identify factors assessed at baseline that were associated with the observed trajectories. In particular, we wanted to identify factors associated with improvement or decline in QoL over time.

## Method

### Design

We used longitudinal data from Times 1 to 3 of the IDEAL cohort study; full details are in the published protocol (Clare, Nelis, et al., 2014). An involvement group of people with dementia and caregivers, known as ALWAYs (Action on Living Well: Asking You), contributed to study design and interpretation of findings. Participants with mild-to-moderate dementia were recruited from August 2014 to July 2016 through memory services and specialist clinics in 29 National Health Service sites throughout England, Scotland, and Wales, and via the Join Dementia Research portal www.joindementiaresearch.nihr.ac.uk/.

Inclusion criteria were a clinical diagnosis of dementia (any subtype), mild-to-moderate cognitive impairment as indicated by Mini-Mental State Examination (Folstein et al., 1975) score ≥15, and living in the community at the time of enrollment. Exclusion criteria were lack of capacity to provide informed consent, presence of terminal illness, and any known risk to researchers conducting home visits. Where possible, a family member or other informal caregiver (hereafter referred to as the “caregiver”) was recruited alongside the person with dementia, both to act as informant and to provide information about experiences of caregiving; however, participation of a caregiver was not mandatory. Participants were interviewed by trained researchers during three home visits at baseline (Time 1; T1; 2014–2016), with follow-up assessments during two home visits 12 (Time 2; T2) and 24 (Time 3; T3) months later. There were 1,545 participants recruited at baseline. Sample size was determined based on the MIDAS (Clare et al., 2012) and Dependence in Alzheimer’s Disease (Jones et al., 2015) studies and to ensure reliability of coefficients based on a proposed analysis using structural equation modeling (Nunnally et al., 1967).

IDEAL was approved by Wales Research Ethics Committee 5 (reference 13/WA/0405) and the Ethics Committee of the School of Psychology, Bangor University (reference 11684) and is registered with UKCRN (registration number 16593). For the present analysis, we used Version 5 of the IDEAL data set. IDEAL T1–T3 data were deposited with the UK data archive in April 2020 and will be available from April 2023. For details of how to access the data, see http://reshare.ukdataservice.ac.uk/854293/.

### Measures

#### Quality of life

QoL was measured with the QoL-AD (Logsdon et al., 2000).

#### Demographic and clinical details and perceived social status

Interviewers collected information on dementia diagnosis, age, sex, education, living situation, and social class. Diagnosis was recorded as Alzheimer’s disease (AD), vascular dementia, mixed AD and vascular dementia, frontotemporal dementia, Parkinson’s disease dementia, dementia with Lewy bodies, or unspecified/other. The MacArthur Scale of Subjective Social Status (Adler et al., 2000) was used to assess perceived social standing, with participants making a rating from 1 (low) to 10 (high). See the Supplementary Appendix for further details.

#### Cognition, functional ability, and awareness

Cognition was assessed with the Addenbrooke’s Cognitive Examination-III (Hsieh et al., 2013), yielding total scores (score range 0–100) and subdomain scores for attention (score range 0–18), memory (score range 0–26), verbal fluency (score range 0–14), language (score range 0–26), and visuospatial ability (score range 0–16). Higher scores indicate better cognitive function. An 11-item amended version of the Functional Activities Questionnaire was used to measure self-rated functional abilities (score range 0–33); higher scores reflect greater impairment (Martyr et al., 2012; Pfeffer et al., 1982). The nine screening questions of the Representations and Adjustment to Dementia Index (Quinn et al., 2018) were used to assess awareness; a score of zero indicates lack of acknowledgement of dementia-related difficulties, reflecting low awareness.

#### Physical health

The Charlson Comorbidity Index age-adjusted score (Charlson et al., 2008) identified the number of chronic conditions. Subjective health was assessed with the question “How would you rate your health in the past four weeks?” with six ordinal response options ranging from very poor to excellent (Bowling, 2005).

#### Social contact and engagement

Social isolation was measured using the six-item Lubben Social Network Scale (score range 0–30; Lubben et al., 2006); higher scores indicate more social contact. Engagement in social activity was measured with the 13-item Cultural Capital scale; higher scores indicate greater engagement (score range 13–65; Thomson, 2004).

#### Psychological health

Depressive symptoms were assessed using the 10-item Geriatric Depression Scale (Almeida & Almeida, 1999); higher scores indicate higher levels of depressive symptoms. Loneliness was measured using the six-item De Jong Gierveld Loneliness Scale (De Jong Gierveld & Van Tilburg, 2010); higher scores indicate greater loneliness. Self-esteem was measured using the 10-item Rosenberg Self-Esteem Scale (Rosenberg, 1965); higher scores indicate greater self-esteem. The six nonfiller items from the Life-Orientation Test-Revised scale (Scheier et al., 1994) were used to measure optimism; higher scores indicate greater optimism.

### Modeling

**Figure 1.**
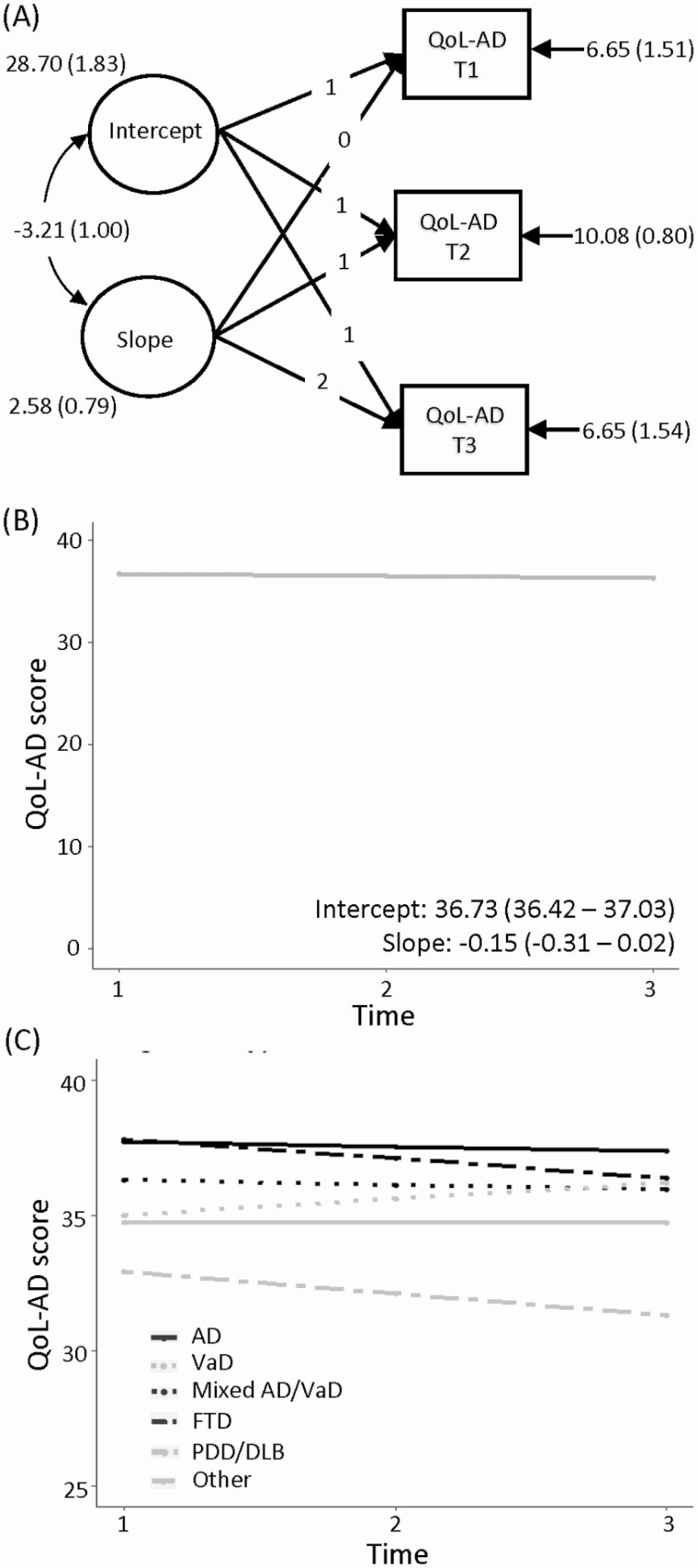
(A) The measurement model for quality of life (QoL). Intercept and slope latent factors are modeled from QoL-AD T1–T3, with the intercept fixed to 1, and the occasions of the slopes fixed to 0, 1, and 2 based on yearly measurement occasions. A linear trend was assumed in view of only having three time points (B). The intercept and trajectory of QoL derived from the model in (A). (C) Visualization of the mean intercepts and slopes for QoL according to diagnosis. AD = Alzheimer’s disease; FTD = frontotemporal dementia; Mixed AD/VaD = Mixed Alzheimer’s and vascular; PDD/DLB = Parkinson’s disease dementia/dementia with Lewy bodies; Other = Unspecified dementia/other; VaD = vascular dementia.

We investigated trajectories of QoL-AD scores with two models operationalized in Mplus v8.2. First, we examined mean change over the three time points using a latent growth curve model (LGCM), comprising a mean intercept and slope, with random effects to account for variation across individuals. The model diagram is shown in Figure 1A. Associations of demographic, cognitive, and psychological covariates measured at baseline with the intercept and slope of QoL were investigated. The second model employed latent class growth analysis (LCGA) and growth mixture modeling (GMM) to examine whether multiple growth trajectories of QoL existed in the sample. Underlying assumptions were tested. The posterior probability of class membership was used to investigate the factors associated with each class in a multinomial regression model. Univariable models incorporated a single predictor, whereas multivariable models incorporated multiple predictors. Models were adjusted for sex, age, and diagnosis, and changes in the intercept and slope were considered significant if 95% confidence intervals did not span 1. Further details are provided in the Supplementary Materials.

In our original cross-sectional modeling, we used a composite measure of “living well” incorporating QoL, satisfaction with life, and well-being (Clare et al., 2019). We measured satisfaction with life using the Satisfaction with Life Scale (SwLS; score range 5–35; Diener et al., 1985), and well-being with the World Health Organization-Five Well-being Index percentage score (WHO-5; score range 0–100; Bech, 2004). To examine whether such a composite measure provides greater explanatory value longitudinally, a latent factor representing “living well” was estimated from QoL-AD, SwLS, and WHO-5 scores. QoL-AD was used as the marker, with the “living well” factor taking on the same scale as QoL-AD. The LGCM and LCGA/GMM models used for QoL-AD were applied to the “living well” latent factor. See Supplementary Appendix, Table S1, and Figure S1.

### Missing Data

Mplus uses the full information maximum likelihood estimator to handle missing data on outcome measures under the assumption that data are missing at random. This assumption was tested and we judged the occurrence of missing data to be ignorable. Multiple imputation of missing data on covariates was generated from Markov Chain Monte Carlo simulations in Mplus. Further details are provided in the Supplementary Appendix and Tables S2 and S3.

## Results

### Cohort Characteristics

There were 1,545 people with dementia recruited to the cohort. Researchers interviewed 1,537 at T1, 1,183 at T2, and 851 at T3. The most common reason for withdrawal was ill-health; death accounted for 48 withdrawals at T2 and 72 at T3. The mean age was 76–77 years and almost two-thirds of participants were male. The distribution of dementia diagnoses, with AD accounting for just over half of all diagnoses, and the proportion of individuals from minority ethnic groups was consistent with British population estimates (Pham et al., 2018; Prince et al., 2014). Mean scores were stable across time for all measures except cognition, which declined. Details are summarized in Table 1.

**Table 1.** Characteristics of People With Dementia in the IDEAL Cohort at T1, T2, and T3, and Scores on Study Variables

Measures		T1	T2	T3
		N	N	N
Took part		1,537	1,183	851
Did not take part at this time point		8	12	—
Died		—	48	72
Withdrew/lost to follow-up		—	302	272
		*N* (%)	*N* (%)	*N* (%)
Diagnosis	AD	851 (55.4%)	661 (55.9%)	488 (57.3%)
	VaD	170 (11.1%)	116 (9.8%)	82 (9.6%)
	Mixed AD/VaD	324 (21.1%)	264 (22.3%)	185 (21.7%)
	FTD	54 (3.5%)	40 (3.4%)	32 (3.8%)
	PDD	44 (2.9%)	34 (2.9%)	17 (2.0%)
	DLB	53 (3.4%)	39 (3.3%)	27 (3.2%)
	Unspecified/other	41 (2.7%)	29 (2.5%)	20 (2.4%)
Demographic details				
Age (years)	<65	134 (8.7%)	89 (7.5%)	67 (7.9%)
	65–69	177 (11.5%)	129 (10.9%)	71 (8.3%)
	70–74	257 (16.7%)	193 (16.3%)	160 (18.8%)
	75–79	367 (23.9%)	269 (22.7%)	171 (20.1%)
	80+	602 (39.2%)	503 (42.5%)	382 (44.9%)
Mean age		76.4 (8.5)	77.2 (8.4)	77.5 (8.5)
Sex	Male	865 (56.3%)	669 (56.6%)	476 (55.9%)
	Female	672 (43.7%)	514 (43.4%)	375 (44.1%)
Education	No qualifications	429 (27.9%)	318 (26.9%)	232 (27.3%)
	School leaving certificate at age 16	271 (17.6%)	197 (16.7%)	136 (16.0%)
	School leaving certificate at age 18	518 (33.7%)	410 (34.7%)	295 (34.7%)
	University	311 (20.2%)	248 (21.0%)	182 (21.4%)
	Missing	8 (0.5%)	10 (0.8%)	6 (0.7%)
Living situation	Lives alone	288 (18.7%)	200 (16.9%)	134 (15.7%)
	Lives with spouse/partner	1,161 (75.5%)	891 (75.3%)	645 (75.4%)
	Lives with other	86 (5.6%)	67 (5.7%)	45 (5.3%)
	Unclassifiable	2 (0.1%)	1 (0.1%)	1 (0.1%)
	In care home	0 (0%)	24 (2.0%)	29 (3.4%)
Social class	I (Professional)	132 (8.6%)	103 (8.8%)	66 (7.8%)
	II (Managerial and technical)	519 (33.8%)	415 (35.3%)	311 (36.5%)
	III-NM (Skilled nonmanual)	298 (19.4%)	216 (18.3%)	151 (17.7%)
	III-M (Skilled manual)	305 (19.8%)	232 (19.6%)	166 (19.5%)
	IV (Partly skilled)	146 (9.5%)	109 (9.2%)	79 (9.3%)
	V (Unskilled)	38 (2.5%)	26 (2.2%)	15 (1.8%)
	Armed forces	21 (1.4%)	15 (1.3%)	12 (1.4%)
	Missing/unclassifiable/NA	78 (5.0%)	67 (5.7%)	51 (6.0%)
Perceived standing in society		6.7 (1.7), *N* = 38	6.5 (1.8), *N* = 119	6.6 (1.8), *N* = 122
		Mean (*SD*), missing	Mean (*SD*), missing	Mean (*SD*), missing
Cognition, functional ability, and awareness				
MMSE		23.2 (3.6), *N* = 71	21.6 (5.1), *N* = 12	20.5 (6.2), *N* = 12
ACE-III total		69.2 (13.1), *N* = 104	66.4 (15.9), *N* = 107	64.6 (17.9), *N* = 111
ACE-III fluency		6.8 (3.1), *N* = 35	6.3 (3.3), *N* = 95	6.2 (3.4), *N* = 110
ACE-III attention		13.9 (3.0), *N* = 38	13.0 (3.5), *N* = 91	12.6 (3.9), *N* = 107
ACE-III visuospatial		12.5 (3.2), *N* = 52	12.2 (3.5), *N* = 101	11.8 (3.8), *N* = 110
ACE-III memory		13.5 (5.4), *N* = 59	12.9 (6.0), *N* = 100	12.7 (6.3), *N* = 110
ACE-III language		22.4 (3.7), *N* = 72	21.9 (4.3), *N* = 104	21.3 (5.0), *N* = 111
PwD-rated functional ability		9.6 (7.7), *N* = 54	8.8 (5.6), *N* = 316	9.4 (5.9), *N* = 200
		Mean (*SD*), missing	Mean (*SD*), missing	Mean (*SD*), missing
Awareness				
Low		83 (5.4%)	63 (5.3%)	60 (7.1%)
Evident		1,337 (87.0%)	986 (83.3%)	679 (79.8%)
Missing		117 (7.6%)	134 (11.3%)	117 (13.7%)
		Mean (*SD*), missing	Mean (*SD*), missing	Mean (*SD*), missing
Physical health				
CCI score^a^		7.0 (2.2), *N* = 107	6.8 (2.0), *N* = 79	6.8 (2.0), *N* = 57
Self-rated health		3.8 (1.2), *N* = 5	3.8 (1.1), *N* = 12	3.9 (1.1), *N* = 16
Social contact and engagement				
Social isolation		15.1 (6.2), *N* = 90	14.8 (6.2), *N* = 108	14.5 (6.3), *N* = 125
Cultural capital		22.9 (5.6), *N* = 86	22.2 (5.5), *N* = 107	21.7 (5.4), *N* = 113
Psychological measures				
Depression		2.7 (2.3), *N* = 169	2.4 (2.3), *N* = 108	2.4 (2.1), *N* = 97
Loneliness		1.4 (1.5), *N* = 102	Not asked	1.4 (1.5), *N* = 88
Self-esteem		29.5 (3.8), *N* = 194	Not asked	Not asked
Optimism		15.0 (3.5), *N* = 113	Not asked	Not asked
Living well measures				
QoL-AD		36.8 (5.9), *N* = 144	37.0 (5.9), *N* = 142	37.0 (5.6), *N* = 136
SwLS		26.1 (6.1), *N* = 43	26.3 (6.1), *N* = 76	26.3 (6.3), *N* = 90
WHO-5		61.0 (20.5), *N* = 26	60.9 (20.6), *N* = 56	61.3 (21.0), *N* = 70

*Notes*: ACE-III Addenbrooke’s Cognitive Examination-III; AD = Alzheimer’s disease; CCI = Charlson Comorbidity Index; DLB = dementia with Lewy bodies; FTD = frontotemporal dementia; IDEAL = Improving the experience of Dementia and Enhancing Active Life; MMSE = Mini-Mental State Examination; NA = not applicable; PDD = Parkinson’s disease dementia; PwD = people with dementia; QoL-AD = Quality of Life in Alzheimer’s Disease; *SD* = standard deviation; SwLS = Satisfaction with Life Scale; VaD = vascular dementia; WHO-5 = World Health Organization-Five Well-being Index.

^a^For the CCI age-adjusted score, where a caregiver was participating alongside the person with dementia, s/he was asked to support completion of this measure. At T2 and T3, the caregiver answered these questions if available and the person with dementia only completed them when no caregiver was involved in the study.

### Mean Intercepts and Slopes for QoL

Despite little change in mean QoL-AD score over time, there was considerable individual variation. We used LGCMs to explore the extent of change in QoL-AD scores over time. The model fitted the data well (comparative fit index = 0.998, root mean square error of approximation = 0.037 [0.00–0.09]). As shown in the unconditional model (Figure 1B), the mean score at baseline was 36.7 and there was little change in the trajectory of QoL-AD (−0.15 units per year).

We investigated the effect of demographic and other variables on the mean intercepts and slopes for QoL-AD. Diagnosis, age, sex, education, living situation, social class, and perceived standing in society were incorporated as indicator variables in the LGCM models (Supplementary Table S4 and Figure S2A). At baseline, there were differences in mean QoL-AD scores for each of these measures except sex. However, there was little effect on the trajectory of QoL-AD, with only small differences based on diagnosis and living situation; compared to those with AD, people with vascular dementia tended to improve slightly across time (Figure 1C), and compared to those living with spouses, people living alone tended to decline across time. All other variables were associated with QoL-AD score at baseline, but there was little evidence of any impact longitudinally. For measures of cognition, functional ability, and awareness, there was no evidence of effects on trajectory (Supplementary Table S4 and Figure S2B). For measures of physical health, there were small impacts, with both lower self-rated health and a higher comorbidity index associated with decline (Supplementary Table S4 and Figure S2C). There was no effect of social isolation or cultural capital (Supplementary Table S4 and Figure S2D), while higher depression and loneliness, and lower self-esteem and optimism, were associated with a small decline in QoL-AD (Supplementary Table S4 and Figure S2E). Similar results were found for the “living well” model (Supplementary Table S5). Calculation of a Reliable Change Index (Evans et al., 1998) indicated that a change of 7.1 was required to be confident that the result was not due to measurement error; therefore, these findings suggest that, when considering mean change in the whole sample, none of the measures had any meaningful influence on the trajectory of QoL.

### Classes of QoL

While mean scores indicated little change over time, interindividual differences in the second-order growth factors were statistically significant, with estimated variances pointing to the existence of variation in both intercept and slope. We therefore investigated heterogeneity in trajectories. Model selection is described in the Supplementary Appendix, Table S6, and Figure S3. Based on model fit indices and interpretability, a four-class model with the variances of the global growth factors constrained across the classes to be equal (growth mixture model-class invariant [GMM-CI]) was selected.

**Figure 2.**
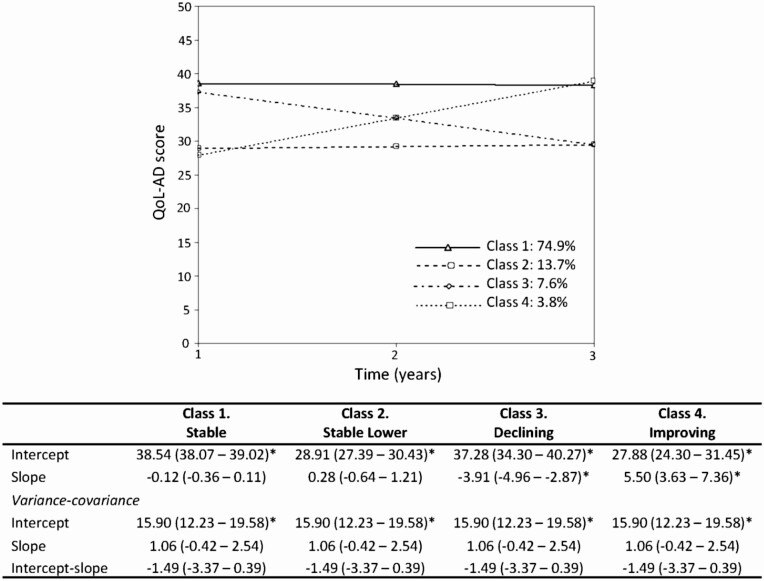
Trajectories of the four classes of quality of life (QoL) determined from the GMM-CI model; Class 1: Stable, Class 2: Stable Lower, Class 3: Declining, Class 4: Improving. The mean intercepts and slopes associated with each class are shown, as are the intercept and slope variances which are equal across classes. 95% confidence intervals are displayed in brackets. * 95% confidence intervals do not span 1.

The resulting four-class solution for QoL-AD had average latent class probabilities ranging from 0.66 to 0.72 (Supplementary Table S7) and an entropy of 0.66, and comprised a stable class (Class 1: hereafter labeled Stable, 74.9%), a stable class with markedly lower QoL scores (Class 2: Stable Lower, 13.7%), a declining class (Class 3: Declining, 7.6%), and an improving class (Class 4: Improving, 3.8%). Trajectories alongside fixed and random effects are shown in Figure 2, and individual participants within each class are plotted in Supplementary Figure S4. The mean decline of 7.8 points in QoL-AD score for the declining class, and the increase of 11 points for the improving class, were considered reliable changes. Additionally, there was a difference of 10 points in mean QoL-AD score at baseline between the Stable and Stable Lower classes which remained across time. Given some uncertainty in class membership, further analyses took into account the probabilities of each individual being a member of each class (see Supplementary Materials). Similar classes were identified for the composite measure of “living well”: Stable (72.0%), Stable Lower (12.7%), Declining (9.9%), and Improving (5.5%) with an entropy of 0.69 (see Supplementary Appendix, Tables S8 and S9, Figures S5–S7).

Characteristics of participants in the four classes are summarized in Table 2. We used multinomial regression to examine associations of study variables with class membership (Table 3). Findings were interpreted using sample statistics alongside odds ratios and their confidence intervals. The Stable class was the reference category. The greatest differences were those between the Stable and the Stable Lower classes; people in the Stable Lower class were more likely to be younger, had increased odds of being diagnosed with vascular or Parkinsonian dementias versus AD, and were less likely to live with a spouse. They were more likely to have no qualifications, be of lower social class, report lower perceived standing in society and cultural capital, be socially isolated, have more comorbidities and poorer self-rated health, and score more negatively on functional abilities, depression, loneliness, self-esteem, and optimism, but did not differ in terms of cognition.

**Table 2.** Characteristics of Each Latent QoL Class

Measures	Class 1. Stable^a^	Class 2. Stable Lower^b^	Class 3. Declining^c^	Class 4. Improving^d^
	*N* (%)	*N* (%)	*N* (%)	*N* (%)
Diagnosis				
AD	653 (58.4%)	84 (41.3%)	59 (51.7%)	24 (42.8%)
VaD	107 (9.6%)	29 (14.1%)	11 (9.8%)	11 (19.7%)
Mixed AD/VaD	238 (21.3%)	47 (22.9%)	25 (21.9%)	12 (21.2%)
FTD	42 (3.7%)	6 (2.9%)	6 (4.9%)	2 (3.3%)
PDD/DLB	55 (4.9%)	29 (14.3%)	11 (9.7%)	6 (10.8%)
Unspecified/other	23 (2.1%)	9 (4.5%)	2 (2.0%)	1 (2.1%)
Demographic details				
Age				
<65	88 (7.9%)	26 (12.9%)	10 (8.4%)	8 (13.6%)
65–69	125 (11.2%)	27 (13.1%)	13 (11.2%)	9 (16.0%)
70–74	187 (16.7%)	38 (18.3%)	20 (17.6%)	8 (14.1%)
75–79	265 (23.8%)	45 (21.9%)	30 (26.1%)	13 (23.2%)
80+	452 (40.4%)	69 (33.7%)	42 (36.6%)	19 (33.1%)
Mean age	76.7 (8.2)	74.8 (9.8)	76.4 (8.3)	74.8 (10.0)
Sex				
Male	742 (66.4%)	138 (67.2%)	78 (68.5%)	41 (72.4%)
Female	376 (33.6%)	67 (32.8%)	36 (31.5%)	16 (27.6%)
Education				
No qualifications	298 (27.0%)	68 (33.6%)	32 (28.7%)	18 (32.4%)
School leaving certificate at age 16	201 (18.2%)	32 (15.6%)	19 (17.2%)	9 (16.0%)
School leaving certificate at age 18	386 (35.0%)	65 (31.9%)	37 (32.8%)	18 (31.4%)
University	219 (19.8%)	38 (18.9%)	24 (21.3%)	11 (20.2%)
Living situation				
Lives alone	202 (18.2%)	46 (22.7%)	24 (20.9%)	8 (14.5%)
Lives with spouse	846 (76.4%)	145 (70.9%)	84 (73.9%)	44 (78.8%)
Lives with others	61 (5.5%)	13 (6.4%)	6 (5.1%)	4 (6.7%)
Social class				
Low (IV/V/armed forces)	134 (12.7%)	37 (19.2%)	14 (13.0%)	9 (17.0%)
Middle (III-NM/III-M)	440 (41.8%)	77 (39.9%)	47 (43.5%)	20 (37.7%)
High (I/II)	478 (45.4%)	79 (40.8%)	47 (43.5%)	24 (45.3%)
	Mean (*SD*)	Mean (*SD*)	Mean (*SD*)	Mean (*SD*)
Standing in society	6.7 (1.7)	5.9 (1.9)	6.6 (1.7)	6.2 (1.9)
Cognition, functional impairment, and awareness				
ACE-III total	69.6 (12.9)	69.1 (13.6)	68.6 (13.2)	67.4 (13.9)
ACE-III fluency	7.0 (3.0)	6.5 (3.1)	6.3 (3.1)	6.3 (3.2)
ACE-III attention	14.0 (2.9)	13.9 (3.1)	13.9 (3.0)	13.2 (3.3)
ACE-III visuospatial	12.8 (3.1)	12.0 (3.5)	12.2 (3.4)	11.6 (3.7)
ACE-III memory	13.4 (5.4)	14.4 (5.3)	13.6 (5.4)	14.0 (5.4)
ACE-III language	22.5 (3.6)	22.3 (3.7)	22.5 (3.6)	22.0 (3.8)
Functional ability	8.6 (7.3)	12.8 (8.2)	10.4 (7.8)	12.5 (8.3)
	*N* (%)	*N* (%)	*N* (%)	*N* (%)
Awareness				
Low	68 (6.6%)	3 (1.6%)	6 (5.3%)	1 (1.1%)
Rest of cohort	959 (93.4%)	169 (98.4%)	100 (94.7%)	51 (98.9%)
	Mean (*SD*)	Mean (*SD*)	Mean (*SD*)	Mean (*SD*)
Physical health				
CCI symptoms	6.9 (2.1)	7.5 (2.5)	7.0 (2.2)	7.2 (2.4)
Self-rated health	4.0 (1.1)	2.9 (1.0)	3.8 (1.2)	3.1 (1.2)
Social contact and engagement				
Social isolation	15.8 (6.1)	12.8 (5.8)	14.8 (6.7)	12.4 (6.0)
Cultural capital	23.4 (5.6)	21.1 (5.3)	22.5 (5.6)	21.2 (5.2)
Psychological measures				
Depression	2.1 (1.9)	5.0 (2.5)	2.8 (2.1)	5.0 (2.6)
Loneliness	1.1 (1.3)	2.4 (1.9)	1.5 (1.4)	2.3 (1.9)
Self-esteem	30.1 (3.5)	26.7 (3.8)	29.0 (3.2)	27.2 (4.2)
Optimism	15.5 (3.2)	12.8 (3.6)	14.6 (3.4)	13.1 (3.9)

*Notes*: ACE-III = Addenbrooke’s Cognitive Examination-III; AD = Alzheimer’s disease; CCI = Charlson Comorbidity Index; DLB = dementia with Lewy bodies; FTD = frontotemporal dementia; PDD = Parkinson’s disease dementia; QoL = quality of life; *SD* = standard deviation; VaD = vascular dementia. Misclassification error derived from the posterior probabilities is taken into account and numbers within each class are rounded to the nearest integer.

^a^ *N* = 1,117, 74.9%.

^b^ *N* = 205, 13.7%.

^c^ *N* = 113, 7.6%.

^d^ *N* = 57, 3.8%.

**Table 3.** Predicting Class Membership for QoL Using Multinomial Logistic Regression, Adjusting for Sex, Age, and Diagnosis Type

a) Univariable models				
Measures	Class 1. Stable	Class 2. Stable Lower	Class 3. Declining	Class 4. Improving
		OR (95% CI)	OR (95% CI)	OR (95% CI)
Demographics				
Diagnosis^a^ (ref: AD)				
VaD	ref	3.63 (1.56–8.34)*	1.32 (0.15–11.73)	4.82 (1.14–20.35)*
Mixed AD/VaD	ref	1.52 (0.64–3.38)	2.07 (0.58–7.43)	2.19 (0.54–8.97)
FTD	ref	0.15 (0.01–46.12)	7.60 (1.42–40.65)*	2.34 (0.16–34.08)
PDD/DLB	ref	14.95 (5.78–38.69)*	6.20 (0.91–42.48)	2.53 (0.08–78.48)
Unspecified/other	ref	10.61 (3.07–36.64)*	2.93 (0.08–90.21)	0.00 (0.00–0.00)
Age^a^ (years)	ref	0.95 (0.91–0.99)*	0.97 (0.92–1.03)	0.95 (0.86–1.05)
Sex^a^ (ref: Female)				
Male	ref	1.23 (0.72–2.13)	2.12 (0.67–6.66)	1.66 (0.48–5.72)
Education (ref: School leaving certificate at age 18)				
No qualifications	ref	3.39 (1.22–9.49)*	1.84 (0.45–7.46)	1.30 (0.06–17.18)
School leaving certificate at age 16	ref	0.86 (0.27–2.76)	0.75 (0.13–4.38)	1.09 (0.04–22.69)
University	ref	0.74 (0.06–8.71)	1.20 (0.30–4.87)	2.48 (0.07–63.64)
Living situation (ref: Spouse)				
Lives alone	ref	3.52 (1.59–7.82)*	1.30 (0.28–5.94)	0.34 (0.08–1.44)
Lives with others	ref	4.33 (1.33–11.64)*	2.33 (0.26–20.78)	0.58 (0.08–4.38)
Social class (ref: High I-Professional/II-Managerial and technical)				
Low (IV-Partly skilled/V-Unskilled/armed forces)	ref	4.39 (1.56–12.36)*	1.50 (0.25–8.93)	0.26 (0.00–20.26)
Middle (III-NM-Skilled nonmanual/III-M-Skilled manual)	ref	1.60 (0.59–4.30)	1.82 (0.58–5.73)	0.62 (0.15–2.53)
Standing in Society	ref	0.36 (0.24–0.53)*	0.77 (0.53–1.10)	1.00 (0.75–1.33)
Cognition, functional ability, and awareness				
ACE-III total	ref	1.00 (0.97–1.03)	0.99 (0.95–1.03)	0.95 (0.90–0.99)*
ACE-III fluency	ref	0.90 (0.71–1.15)	0.78 (0.55–1.12)	0.97 (0.51–1.85)
ACE-III attention	ref	1.11 (0.94–1.30)	1.01 (0.83–1.24)	0.75 (0.58–0.98)*
ACE-III visuospatial	ref	0.99 (0.77–1.27)	0.90 (0.76–1.04)	0.77 (0.65–0.92)*
ACE-III memory	ref	1.02 (0.97–1.09)	1.03 (0.94–1.14)	0.98 (0.89–1.08)
ACE-III language	ref	0.89 (0.79–1.01)	1.08 (0.85–1.37)	0.97 (0.70–1.37)
Functional ability	ref	1.13 (1.08–1.19)*	1.08 (0.98–1.19)	1.08 (1.00–1.17)*
Low awareness	ref	0.13 (0.01–2.62)	0.58 (0.02–8.35)	0.04 (0.00–2.16)
Physical health				
CCI symptoms	ref	1.80 (1.36–2.39)*	1.16 (0.65–2.07)	1.27 (0.90–1.79)
Self-rated health	ref	0.14 (0.08–0.26)*	0.76 (0.20–2.94)	0.28 (0.07–1.14)
Social contact and engagement				
Social isolation	ref	0.79 (0.69–0.91)*	0.99 (0.75–1.31)	0.86 (0.78–0.96)*
Cultural capital	ref	0.78 (0.70–0.86)*	0.92 (0.80–1.05)	0.94 (0.75–1.18)
Psychological measures				
Depression	ref	4.50 (2.89–7.00)*	1.87 (1.23–2.84)*	3.65 (1.91–6.98)*
Loneliness	ref	3.29 (2.40–4.52)*	1.81 (1.13–2.92)*	2.70 (1.63–4.47)*
Self-esteem	ref	0.52 (0.39–0.69)*	0.72 (0.59–0.88)*	0.59 (0.23–1.52)
Optimism	ref	0.63 (0.49–0.80)*	0.77 (0.65–0.93)*	0.75 (0.51–1.10)
b) Multivariable model				
Depression	ref	3.52 (1.95–5.76)*	1.47 (0.90–2.28)	2.94 (1.16–7.47)*
Loneliness	ref	1.84 (1.15–2.96)*	1.37 (0.83–2.25)	1.93 (0.87–4.27)
Self-esteem	ref	0.76 (0.57–1.02)	0.87 (0.66–1.11)	0.89 (0.59–1.34)
Optimism	ref	0.99 (0.73–1.35)	0.91 (0.71–1.20)	1.02 (0.65–1.60)

*Notes*: ACE-III = Addenbrooke’s Cognitive Examination-III; AD = Alzheimer’s disease; CCI = Charlson Comorbidity Index; CI = confidence intervals; DLB = dementia with Lewy bodies; FTD = frontotemporal dementia; OR = odds ratio; PDD = Parkinson’s disease dementia; QoL = quality of life; ref = reference category/class; VaD = vascular dementia. Reference class is Class 1: Stable. Class membership error is accounted for.

^a^Unadjusted.

*95% confidence intervals do not span 1.

The Improving class had similar baseline QoL-AD scores to the Stable Lower class. For measures of functional ability, social isolation, depression, and loneliness, findings were similar to those for the Stable Lower class. Compared to the Stable class, the Improving class was more likely to have lower baseline levels of cognition and, although confidence intervals were wide due to small numbers, there was a greater likelihood of being diagnosed with vascular dementia relative to AD.

The Declining class had similar baseline QoL-AD scores to the Stable group. There were increased proportions of people with rarer dementia subtypes (frontotemporal dementia, Parkinson’s disease dementia, and dementia with Lewy bodies) in the Declining class compared to the Stable class, and for frontotemporal dementia this was supported in the multinomial regression despite the small sample sizes. Higher baseline levels of depression and loneliness, and lower levels of self-esteem and optimism were associated with greater likelihood of being in the Declining class despite baseline QoL-AD scores being commensurate with the Stable class. When entered into a multivariable model, these psychological measures were not independently associated with decline in QoL. However, depression remained independently associated with the Improving class, and both depression and loneliness with the Stable Lower class.

Similar findings were observed when using the broader composite measure of “living well” incorporating QoL, well-being, and satisfaction with life, except that in addition greater functional impairment at baseline was associated with membership of the Declining class and poorer physical health with membership of the Improving class, as shown in Supplementary Tables S10 and S11.

## Discussion

We modeled longitudinal change in self-rated QoL in a cohort of community-dwelling individuals with mild-to-moderate dementia in Britain, followed up after 12 and 24 months. Mean QoL-AD scores were stable over time, with a negligible nonsignificant annual decline of 0.15 points. This masked distinct QoL trajectories. Most participants remained stable, and could be differentiated into Stable (74.9%) and Stable Lower (13.7%) classes. Compared to the Stable class, the Stable Lower class was characterized by lower social status, poorer physical health, and lower baseline scores on all measures except cognition. There were two smaller classes, one with low baseline scores and an improving trajectory (3.8%) and one with higher baseline scores and a declining trajectory (7.6%). Compared to the Stable Lower class, the Improving class had lower baseline cognitive test scores. Compared to the Stable class, the Declining class had higher baseline levels of depression and loneliness and lower levels of self-esteem and optimism. Although numbers were small, the proportion of people with rarer types of dementia appeared higher in the Declining class. Incorporating measures of satisfaction with life and well-being into the models alongside QoL produced similar results. These findings must be interpreted cautiously, but suggest the potential for a more nuanced approach to supporting QoL in people with mild-to-moderate dementia.

The finding of no group-level change is consistent with most previous studies (Andrieu et al., 2016; Bosboom & Almeida, 2016; Clare et al., 2012; Clare, Woods, et al., 2014; Conde-Sala et al., 2016; Dourado et al., 2016; Hongisto et al., 2015; Livingston et al., 2008; O’Shea et al., 2020; Sousa et al., 2018; Trigg et al., 2015). Kisvetrová et al. (2020) reported a mean decline of 1.98 points on the QoL-AD at 24 months, but this was much smaller than the reliable change index calculated for our sample. Our study provides robust confirmatory evidence as it addressed key limitations of earlier studies.

In the context of no overall change, previous studies have noted individual variability (Livingston et al., 2008; Trigg et al., 2015), but few have explored evidence for different QoL trajectories. The current study supports our previous findings (Clare, Nelis, et al., 2014) of a large proportion of people with stable trajectories and smaller proportions with improving and declining trajectories, but in a much larger sample; additionally, we were able for the first time to identify a stable subgroup with lower baseline QoL scores. This Stable Lower group demonstrates the association with poor QoL of a broad range of factors, including disadvantage related to social structure, poor physical health, functional disability, and low psychological well-being, confirming indications observed in cross-sectional modeling (Clare et al., 2019). The only factor distinguishing the Improving group from the Stable Lower group was lower cognitive test scores; we can only speculate about the reasons for improvement in QoL ratings, but perhaps, due to declining cognitive ability, this group received more support, or became more accepting of limitations over time. For those with better baseline QoL, poorer psychological well-being appeared to be the key driver of decline.

The availability of data from three time points is a strength since most previous studies included only one follow-up, but given the degree of individual variation, additional follow-ups would allow deeper exploration; this will be attempted as further data from the IDEAL cohort become available, albeit with reduced numbers due to attrition. Attrition levels over the three time points included here were relatively high, but this is unsurprising for a cohort of people with dementia. Participants were recruited on the basis of attendance at British memory clinics, and while the proportion of people from minority ethnic groups was consistent with British population estimates, numbers were small; a more culturally diverse sample might yield different results. Similarly, the proportions diagnosed with rarer types of dementia were consistent with population estimates, but numbers in these subgroups were small, and further work would be needed to establish whether current findings hold within these groups.

The classes extracted from the GMM-CI model and the results of the multinomial regression should be interpreted with caution as GMM is an exploratory approach and findings vary based on model specification. The GMM with free variances both across and within classes is optimal, but the literature indicates that these models are fraught with convergence issues and inadmissible solutions (Diallo et al., 2016; McNeish & Harring, 2021). To support convergence it was necessary to apply constraints on the model, in this case constraining the intercept and slope variances to be equal across classes. However, individuals are then classified while satisfying the within-class growth characteristics defined by the model and this can result in errors in enumeration of classes, in the classification of individuals and in parameter estimates. Given that the data include only three time points, which means a linear trend must be assumed, and that there was approximately 45% attrition from T1 to T3, the constrained GMM is the best model that can be achieved with our data. It is notable that while there may be some uncertainty in the extracted classes with this approach, the classes correspond with the declining, improving, and stable groups identified in our previous study of QoL trajectories in a smaller cohort of people with dementia (Clare et al., 2014). In addition, compared to the Stable group the findings show strong associations between poorer scores on psychological, physical, and social measures and membership of the Stable Lower group, as would be expected given that this group score more poorly on QoL. Furthermore, we have extracted the individuals within the classes and plotted their data, and there are clear distinctions in the patterns of trajectories. Finally, QoL is a diffuse construct and measures based on a broad conceptualization, such as the QoL-AD, ask about various aspects of people’s lives, including cognition, physical health, mood, and relationships, as well as how people feel about their life as a whole; hence, there are overlaps with some predictor variables.

The study has implications for how we understand and use measures of QoL. Although such measures are often employed to assess outcomes, they contain no indication of what constitutes meaningful change; our analyses suggest that the magnitude of change required may be considerably greater than is often assumed. The encouraging finding that most people with mild-to-moderate dementia had relatively high QoL scores that remained stable over time should be taken into consideration when using QoL measures as indicators of outcome in intervention studies.

The most important implications, however, arise because elucidating different trajectories creates potential for more personalized and contextualized approaches to supporting QoL, focusing on improving QoL for those with low baseline scores and on maintaining QoL and preventing decline for those with higher baseline scores. For people with higher baseline scores, our findings suggest that psychological well-being should be the main focus of efforts to support QoL. Alongside appropriate medication (Dou et al., 2018), psychosocial approaches and introduction of dementia-friendly environments can underpin these efforts. For example, psychological well-being can be enhanced through peer support (Leung et al., 2015) and participation in enjoyable activities (Logsdon et al., 2007), while nonpharmacological interventions may be helpful in treating depression (Orgeta et al., 2015). For people with lower baseline scores, the profile suggests the intersection of multiple sources of disadvantage, and hence offers potential avenues for improving QoL. These include addressing structural issues such as social isolation as well as supporting functional ability and mood. The finding of an improving trajectory shows that improvement is possible for some. Finally, cognition was not associated with decline in QoL. Cognitive training interventions are, therefore, unlikely to improve QoL, supporting the view that they should not be recommended on these grounds (National Institute for Health and Clinical Excellence, 2018).

## Conclusion

This study demonstrates for the first time in a large sample of community-dwelling individuals with mild-to-moderate dementia the presence of groups with different levels and trajectories of QoL, while confirming previous observations of stability in mean score over time for the sample as a whole. Identification of factors associated with different trajectories suggests that efforts to prevent decline in QoL should be focused on supporting those people experiencing low mood or depression, while efforts to improve QoL for low-scoring individuals should additionally address functional ability, social isolation, and disadvantage related to social structure. Understanding the level of QoL experienced by, and the factors salient for, each person with dementia, is an important element in personalized care planning. Applying this understanding from the time of diagnosis can help to maintain or improve, and prevent decline in, QoL.

## Supplementary Material

gbac022_suppl_Supplementary_Material
