# Supplementary material for: Longitudinal Trajectories of Quality of Life Among People With Mild-to-Moderate Dementia: A Latent Growth Model Approach With IDEAL Cohort Study Data
Source: J Gerontol B Psychol Sci Soc Sci. 2022 Feb 3;77(6):1037–50. doi: 10.1093/geronb/gbac022 (PMC9159063; doi:10.1093/geronb/gbac022)
Supplement: gbac022_suppl_Supplementary_Material [file gbac022_suppl_supplementary_material.pdf]

Longitudinal trajectories of quality of life among people with mild-to-moderate dementia: a latent growth model approach with IDEAL cohort study data

Supplementary Material

Supplementary Appendix

Additional information about diagnosis categories, demographic variables, and other measures.

Dementia sub-type was obtained from medical records and classified into seven groups as detailed in the main text; ‘other’ covers additional rare sub-types or mixed diagnoses. For analysis purposes, PDD and DLB were combined. Where a participant’s diagnosis changed throughout the course of the study, the last known diagnosis was used.

Social class was measured using the UK National Statistics Socio-Economic Classification which is based on main occupation (Office for National Statistics, 2010) and is the standard UK classification that groups individuals into seven groups: I (Professional), II (Managerial and technical), III-NM (Skilled non-manual), III-M (Skilled manual), IV (Partly skilled), V (Unskilled), and armed forces. These categories were condensed into three subgroups for analysis; high (I/II), middle (III-NM/III-M) and low (IV/V/armed forces). Those who did not fall into one of these categories were considered missing, including those who were unclassifiable or those who selected ‘not applicable’.

Educational qualifications were categorised into four groups: no qualification, school leaving certificate at 16, school leaving certificate at 18, and university-level qualification. Living situation was classified into four groups: living alone, living with spouse/partner, living with others and unclassifiable, as described in previous analyses of IDEAL cohort data (Clare et al., 2020).

With regard to the measure of awareness, the small number of participants who scored zero on screening but were administered the rest of the RADIX in error were treated as missing.

Latent Growth Curve Model

To determine how quality of life and indices of 'living well' change over time for people with dementia, latent growth curve growth modelling (LGCM) was conducted in Mplus Version 8.2 (Muthén & Muthén, 1998-2017) using the first three waves of IDEAL data (T1-T3). A LGCM consists of a measurement model which is then extended to a second order growth model allowing estimation of the mean intercept (baseline) and the mean slope (change over time) in the variable of interest, with random effects to account for variation across individuals (Wickrama et al., 2016).

The measurement model for quality of life as indicated by scores on the QoL-AD scale is described in the main paper. Here we describe the model for 'living well'. We built the 'living well' latent factor from three measures, QoL-AD, Satisfaction with Life Scale (SwLS), and the WHO-5 Well-Being Index (WHO-5), at each time-point by longitudinal confirmatory factor analysis (LCFA). QoL-AD was selected as the marker variable, with loading fixed to 1 at each time point and the intercept fixed to zero to allow for model identification. The scale for 'living well' was expressed on the same scale as QoL-AD and the variance of each latent factor and covariance among latent factors were defined by QoL-AD (Brown 2006). The associations between SwLS and WHO-5 were estimated relative to their association with QoL-AD. Variances were estimated for each subdomain indicator and autocorrelated errors specified and retained in the model to avoid misspecification (Little, 2013). For a model to be considered good, a Comparative Fit index (CFI) and Tucker-Lewis index (TLI) greater than 0.90 and a root mean square error of approximation (RMSEA) less than 0.08 (Hu & Bentler, 1999) are required. As shown in Supplementary Table S1, the

unconstrained measurement model (configural model) was a good fit to the data indicating that each factor was defined by the same variables and that the same general pattern of factor loadings held across time (Millsap & Cham, 2012; Millsap & Olivera-Aguilar, 2012). In order for meaningful comparisons to be made in a LCFA, the assumption of longitudinal measurement variance should be met (Byrne & Watkins, 2003; Chen, 2007). Three levels of measurement invariance were tested imposing additional restrictions at each step: metric invariance (constrained factor loadings across measurement occasions), scalar invariance (constrained factor loadings across measurement occasions and intercepts across time to be equal), and strict invariance (constrained factor loadings across measurement occasions, and intercepts and residual variances across time to be equal). Each level of measurement invariance was applied and model fit indices examined to ensure that the model fit did not weaken when each level of constraint was applied. Studies have suggested that CFI, RMSEA and SRMR are the most important indicators when it comes to testing measurement invariance, with strict cut offs of <0.01 change in CFI, <0.015 change in RMSEA, and <0.030 change in SRMR (Chen, 2007; Cheung & Rensvold, 2002). ΔX^2 was also examined but as it is sensitive to sample size it was not relied upon (Chen, 2007; Kline, 2011; Schermelleh-Engel et al., 2003). As shown in Supplementary Table S1, each more constricted model had minimal impact on fit indices when compared with the previous model, indicating that metric, scalar and strict measurement invariance held. Further analyses were conducted with the strict invariance model.

The ‘living well’ factors defined in the LCFA model were used as indicators of the second-order growth curve where the intercept and slope factors of ‘living well’ were estimated, each with their associated mean and variance. The model diagram is shown in Supplementary Figure S1A. The equivalent measurement model for quality of life using only QoL-AD measurements at T1-T3 as indicators of the second-order growth curve is shown in Figure 1A in the main paper. The intercept loadings were fixed to 1 for each latent intercept,

and 0, 1 and 2 for time based on the yearly measurement occasions. Due to only having 3 time points a linear trend was assumed.

Missing data

Where there are missing values on outcome measures, Mplus uses the full information maximum likelihood (FIML) estimator (Enders & Bandalos, 2001), which allows computation of parameter estimates on the basis of all available data without imputing or dropping data when missing under the assumption that data are missing at random (MAR). To test this assumption, we conducted growth curve analyses for QoL-AD, SwLS and WHO-5 and compared the estimates with a Diggle-Kenward selection model that incorporates dropout (Diggle & Kenward, 1994; Muthen et al., 2011) (Supplementary Table S2); we judged the occurrence of missing data to be ignorable. To specifically test whether death was an informative missing process, we adopted a joint model approach within the JM package in R, which allows simultaneous modelling of longitudinal and time to event data (Rouanet et al., 2019) using mixed models and a Cox proportional hazards regression model. There was minimal impact on the estimates compared to the model without death (Supplementary Table S3). Multiple imputation of missing data on covariates was generated from Markov Chain Monte Carlo (MCMC) simulations in Mplus (Asparouhov & Muthen, 2010). Estimates from 25 imputed datasets were combined using Rubin’s rules (Rubin, 1996).

Latent class growth analysis and growth mixture modelling

So far, our approach has assumed that growth trajectories of all individuals can be adequately described using a single estimate of growth parameters. We employed latent class growth analysis (LCGA) and growth mixture modelling (GMM) to examine whether multiple growth trajectories of quality of life and ‘living well’ exist in the IDEAL cohort population (Jung & Wickrama, 2008; Muthen, 2004; Muthen & Shedden, 1999). Different assumptions were tested. The LCGA fixes variances of the global growth factors to zero across classes (assuming trajectories within a class are homogeneous). The GMM-CI constrains the

variances of the global growth factors across classes to be equal (homogeneous classes), and the GMM-CV freely estimates all variances of the global growth factors across classes. A frequent problem with these models is non-convergence and local solutions (Hipp & Bauer, 2006), with the more complex models, particularly those with free variances, more likely to experience convergence difficulties (Grimm & Ram, 2009). To support convergence, residual variances were free across time but constrained across classes. Between 1 and 5 class solutions were tested for each assumption, with 1000 random starts and 20 iterations for each model in order to avoid local solutions. Following successful convergence, the optimal number of distinct trajectories were determined using BIC, sample size adjusted BIC (ssBIC), the Lo-Mendell-Rubin likelihood ratio test (LMR-LRT) and the bootstrapped likelihood ratio test (BLRT) which provide between model comparisons (k vs k-1), and entropy (Jung & Wickrama, 2008; Nylund et al., 2007; Tein et al., 2013). Entropy is a standardized index of model-based classification accuracy based on the average posterior probability, with higher values indicating clearer class separation (Muthen, 2004). Substantive criteria were based on a class size greater than 1% and theoretical and practical interpretability of the classes. Upon finding an optimal solution, the model was repeated with double the number of starts and iterations to ensure a global solution.

Due to its person-centred approach, GMMs allow for examination of predictors of class membership (Wickrama et al., 2016). The categorical latent class is related to the covariates by way of multinomial logistic regression which assigns each individual fractionally to all classes using posterior probabilities. Predictors of class were examined using the '3-step' approach in Mplus (R3STEP) in order to protect the latent class structure from influences of the covariates (Asparouhov & Muthen, 2013; Vermunt, 2010).

Latent Trajectory Model selection

Three models were tested as described above: LCGA, GMM-CI and GMM-CV. For each model we ran 1 to 5 class solutions. Results for quality of life are displayed in

Supplementary Table S6 and the associated plots in Supplementary Figure S3. Results for ‘living well’ are displayed in Supplementary Table S8 and the associated plots in Supplementary Figure S5. The LCGA is less computationally intensive and all classes ran with no convergence issues. For the GMM-CI the 5-class model produced an inadmissible solution, as did 3-class, 4-class and 5-class models of the GMM-CV. Given all available information including model fit indices, interpretability and theoretical considerations, the optimal model that converged and produced an admissible solution was the 4-class GMM-CI for both quality of life and ‘living well’. Its results were most consistent with a good model fit, having the lowest ssBIC, and significant LMR-LRT and BLRT values suggested this model was an improvement over the 3-model solution.

Supplementary Tables and Figures

Supplementary Table S1. Testing factorial measurement invariance for the 'living well' latent factor. The configural model has no constraints. The metric model has constrained factor loadings across each measurement occasion. The scalar model has constrained factor loadings across each measurement occasion and intercepts across time are set to be equal. The strict model has constrained factor loadings across measurement occasions, and intercepts and variances across time are set to be equal. ΔCFI, ΔRMSEA and ΔSRMR were determined to assess model fit. ΔX^2 was examined but not relied upon.

**Model**	**BIC**	**X^2(df)**	**CFI**	**TLI**	**RMSEA (95% CI)**	**SRMR**	**ΔX^2(Δdf)/p**	**ΔCFI**	**ΔTLI**	**ΔRMSEA**	**ΔSRMR**
Configural	66256	25.18(15)	0.998	0.996	0.021 (0.002 – 0.035)	0.015					
Metric	66239	36.75(19)	0.997	0.995	0.025 (0.012 – 0.036)	0.033	11.57(4)/0.021	0.001	0.001	0.004	0.018
Scalar	66212	39.92(23)	0.997	0.996	0.022 (0.009 – 0.033)	0.031	3.17(4)/0.530	0.000	0.001	0.003	0.002
Strict	66184	55.81(29)	0.996	0.995	0.025 (0.015 – 0.034)	0.041	15.89(6)/0.014	0.001	0.001	0.003	0.010

Note. Bayesian Information Criterion, BIC; degrees of freedom, df; comparative fit index, CFI; Tucker-Lewis index, TLI; root mean square error of approximation, RMSEA; standardized root mean squared residual, SRMR.

Supplementary Table S2. A Diggle-Kenward selection model, which is an extension of the growth model taking into account dropout, was conducted on QoL-AD, SwLS and WHO-5 to determine whether dropout impacted on the estimates. An unconditional model was assessed, as were models incorporating sex, age or diagnosis. Estimates for the intercept and slope are presented alongside standard errors.

**Growth factors**	**Unconditional**		**Sex**		**Age**		**Diagnosis Type**	
	**MAR model (est, SE)**	**DK Model (est, SE)**	**MAR model (est, SE)**	**DK Model (est, SE)**	**MAR model (est, SE)**	**DK Model (est, SE)**	**MAR model (est, SE)**	**DK Model (est, SE)**
QoL-AD								
Intercept	36.73 (0.16)	36.72 (0.16)	36.88 (0.24)	36.88 (0.24)	31.56 (1.38)	31.71 (1.39)	37.72 (0.20)	37.70 (0.20)
Slope	-0.15 (0.09)	0.03 (0.26)	-0.07 (0.13)	0.00 (0.23)	0.57 (0.75)	-0.10 (0.81)	-0.18 (0.11)	-0.16 (0.19)
Male Intercept	-	-	-0.29 (0.31)	-0.30 (0.31)	-	-	-	-
Male Slope	-	-	-0.13 (0.17)	-0.13 (0.17)	-	-	-	-
Age Intercept	-	-	-	-	0.07 (0.02)	0.07 (0.02)	-	-
Age Slope	-	-	-	-	-0.01 (0.01)	0.02 (0.01)	-	-
VaD Intercept (ref: AD)	-	-	-	-	-	-	-2.70 (0.51)	-2.72 (0.51)

Mixed AD/VaD Intercept (ref: AD)	-	-	-	-	-	-	-1.41 (0.38)	-1.39 (0.38)
FTD Intercept (ref: AD)	-	-	-	-	-	-	0.09 (0.81)	0.07 (0.81)
PDD/DLB Intercept (ref: AD)	-	-	-	-	-	-	-4.80 (0.62)	-4.78 (0.62)
Other Intercept (ref: AD)	-	-	-	-	-	-	-3.00 (1.01)	-3.01 (1.00)
VaD Slope (ref: AD)	-	-	-	-	-	-	0.76 (0.30)	0.74 (0.31)
Mixed Slope (ref: AD)	-	-	-	-	-	-	-0.00 (0.21)	-0.01 (0.21)
FTD Slope (ref: AD)	-	-	-	-	-	-	-0.54 (0.44)	-0.56 (0.44)
PDD/DLB Slope (ref: AD)	-	-	-	-	-	-	-0.62 (0.38)	-0.60 (0.40)

Other Slope (ref: AD)	-	-	-	-	-	-	0.18 (0.57)	0.17 (0.57)
SwLS								
Intercept	26.09 (0.15)	26.09 (0.15)	25.99 (0.23)	25.98 (0.23)	17.77 (1.37)	17.69 (1.37)	26.90 (0.20)	26.90 (0.20)
Slope	-0.07 (0.09)	-0.11 (0.18)	-0.00 (0.14)	0.04 (0.22)	-0.85 (0.81)	-0.96 (1.00)	-0.15 (0.12)	-0.22 (0.18)
Male Intercept	-	-	0.18 (0.31)	0.18 (0.31)				
Male Slope	-	-	-0.12 (0.18)	-0.13 (0.18)				
Age Intercept	-	-	-	-	0.11 (0.02)	0.11 (0.02)		
Age Slope	-	-	-	-	0.01 (0.01)	0.04 (0.01)		
VaD Intercept (ref: AD)	-	-	-	-	-	-	-2.11 (0.50)	-2.12 (0.50)
Mixed AD/VaD Intercept (ref: AD)	-	-	-	-	-	-	-0.91 (0.38)	-0.91 (0.38)
FTD Intercept (ref: AD)	-	-	-	-	-	-	-1.56 (0.81)	-1.59 (0.81)
PDD/DLB Intercept	-	-	-	-	-	-	-4.36 (0.61)	-4.34 (0.61)

(ref: AD)								
Other Intercept	-	-	-	-	-	-	-1.53 (0.99)	-1.53 (0.99)
(ref: AD)								
VaD Slope	-	-	-	-	-	-	0.26 (0.31)	0.28 (0.31)
(ref: AD)								
Mixed Slope	-	-	-	-	-	-	0.33 (0.23)	0.34 (0.23)
(ref: AD)								
FTD Slope	-	-	-	-	-	-	-0.68 (0.49)	-0.67 (0.48)
(ref: AD)								
PDD/DLB Slope	-	-	-	-	-	-	0.09 (0.41)	0.06 (0.42)
(ref: AD)								
Other Slope	-	-	-	-	-	-	-0.21 (0.61)	-0.18 (0.61)
(ref: AD)								
WHO-5								
Intercept	15.21 (0.13)	15.21 (0.13)	14.99 (0.20)	15.00 (0.20)	9.63 (1.16)	9.86 (1.17)	15.96 (0.17)	15.96 (0.17)
Slope	-0.10 (0.08)	-0.11 (0.21)	-0.08 (0.12)	0.04 (0.30)	0.93 (0.71)	-0.23 (0.83)	-0.15 (0.10)	-0.24 (0.18)

Male Intercept	-	-	-0.37 (0.26)	-0.37 (0.26)	-	-	-	-
Male Slope	-	-	-0.03 (0.16)	-0.04 (0.16)	-	-	-	-
Age Intercept	-	-	-	-	0.07 (0.02)	0.07 (0.02)	-	-
Age Slope	-	-	-	-	-0.01 (0.01)	0.02 (0.01)	-	-
VaD Intercept (ref: AD)	-	-	-	-	-	-	-1.98 (0.43)	-2.00 (0.43)
Mixed AD/VaD Intercept (ref: AD)	-	-	-	-	-	-	-1.06 (0.32)	-1.05 (0.32)
FTD Intercept (ref: AD)	-	-	-	-	-	-	-0.64 (0.68)	-0.67 (0.68)
PDD/DLB Intercept (ref: AD)	-	-	-	-	-	-	-3.80 (0.51)	-3.79 (0.51)
Other Intercept (ref: AD)	-	-	-	-	-	-	-1.20 (0.83)	-1.19 (0.83)
VaD Slope (ref: AD)	-	-	-	-	-	-	0.52 (0.27)	0.47 (0.27)

Mixed Slope (ref: AD)	-	-	-	-	-	-	0.01 (0.20)	-0.01 (0.20)
FTD Slope (ref: AD)	-	-	-	-	-	-	-0.44 (0.42)	-0.43 (0.42)
PDD/DLB Slope (ref: AD)	-	-	-	-	-	-	-0.11 (0.35)	-0.17 (0.38)
Other Slope (ref: AD)	-	-	-	-	-	-	0.32 (0.54)	0.27 (0.54)

Note: estimate, est; standard error; SE; reference category, ref; Alzheimer's disease, AD; vascular dementia, VaD; mixed Alzheimer's and vascular, Mixed AD/VaD; frontotemporal dementia, FTD; Parkinson's disease dementia/dementia with Lewy bodies, PDD/DLB; Unspecified dementia/Other, Other; missing at random model, MAR model; Diggle-Kenward selection model, DK model; Quality of Life in Alzheimer's Disease, QoL-AD; Satisfaction with life scale, SwLS; World Health Organization-Five Well-being Index, WHO-5.

Supplementary Table S3. A joint model was conducted for QoL-AD, SwLS and WHO-5 to determine whether death impacted on the estimates. An unconditional model was assessed, as were models incorporating sex or diagnosis. Estimates for the intercept and slope are presented alongside standard errors. The joint model incorporates a mixed-effects model with a Cox proportional hazards regression model.

**Growth factors**	**Unconditional**		**Sex**		**Diagnosis Type**	
	**Mixed Model (est, SE)**	**Joint Model (est, SE)**	**Mixed Model (est, SE)**	**Joint Model (est, SE)**	**Mixed Model (est, SE)**	**Joint Model (est, SE)**
QoL-AD						
Intercept	36.89 (0.20)	36.49 (0.21)	36.98 (0.31)	36.69 (0.25)	37.90 (0.27)	37.95 (0.27)
Slope	-0.15 (0.08)	-0.15 (0.09)	-0.07 (0.13)	-0.09 (0.11)	-0.18 (0.11)	-0.17 (0.11)
Male Intercept	-	-	-0.16 (0.41)	-0.14 (0.44)	-	-
Male Slope	-	-	-0.13 (0.17)	-0.13 (0.18)	-	-
VaD Intercept (ref: AD)	-	-	-	-	-3.47 (0.68)	-3.22 (0.68)
Mixed AD/VaD Intercept (ref: AD)	-	-	-	-	-1.40 (0.51)	-1.44 (0.53)
FTD Intercept (ref: AD)	-	-	-	-	0.55 (1.07)	0.75 (1.02)
PDD/DLB Intercept (ref: AD)	-	-	-	-	-4.21 (0.86)	-4.38 (0.90)
Other Intercept (ref: AD)	-	-	-	-	-3.20 (1.36)	-3.01 (1.42)
VaD Slope	-	-	-	-	0.75 (0.30)	0.80 (0.30)

(ref: AD)						
Mixed Slope	-	-	-	-	-0.00 (0.21)	-0.04 (0.22)
(ref: AD)						
FTD Slope	-	-	-	-	-0.55 (0.43)	-0.54 (0.44)
(ref: AD)						
PDD/DLB Slope	-	-	-	-	-0.63 (0.38)	-0.70 (0.40)
(ref: AD)						
Other Slope	-	-	-	-	0.17 (0.57)	0.22 (0.62)
(ref: AD)						
SwLS						
Intercept	26.16 (0.20)	26.06 (0.21)	26.00 (0.31)	25.95 (0.25)	27.06 (0.27)	27.03 (0.28)
Slope	-0.07 (0.09)	-0.08 (0.10)	-0.00 (0.14)	-0.05 (0.11)	-0.15 (0.12)	-0.15 (0.12)
Male Intercept	-	-	0.28 (0.41)	0.33 (0.44)	-	-
Male Slope	-	-	-0.12 (0.18)	-0.07 (0.19)	-	-
VaD Intercept	-	-	-	-	-2.36 (0.68)	-2.34 (0.68)
(ref: AD)						
Mixed AD/VaD	-	-	-	-	-1.24 (0.68)	-1.21 (0.69)
Intercept						
(ref: AD)						
FTD Intercept	-	-	-	-	-0.89 (1.08)	-0.92 (1.05)
(ref: AD)						
PDD/DLB Intercept	-	-	-	-	-4.45 (0.85)	-4.28 (0.86)
(ref: AD)						
Other Intercept	-	-	-	-	-1.33 (1.34)	-1.11 (1.32)
(ref: AD)						
VaD Slope	-	-	-	-	0.26 (0.31)	0.26 (0.31)

(ref: AD)						
Mixed Slope (ref: AD)	-	-	-	-	0.33 (0.23)	0.31 (0.23)
FTD Slope (ref: AD)	-	-	-	-	-0.68 (0.49)	-0.71 (0.48)
PDD/DLB Slope (ref: AD)	-	-	-	-	0.09 (0.41)	0.12 (0.41)
Other Slope (ref: AD)	-	-	-	-	-0.21 (0.61)	-0.20 (0.61)
WHO-5						
Intercept	15.32 (0.18)	15.30 (0.18)	15.11 (0.27)	15.42 (0.21)	16.11 (0.23)	16.11 (0.23)
Slope	-0.10 (0.08)	-0.11 (0.08)	-0.09 (0.12)	-0.14 (0.09)	-0.16 (0.10)	-0.16 (0.10)
Male Intercept	-	-	0.38 (0.36)	0.37 (0.38)	-	-
Male Slope	-	-	-0.03 (0.16)	-0.07 (0.17)	-	-
VaD Intercept (ref: AD)	-	-	-	-	-2.40 (0.59)	-2.50 (0.58)
Mixed AD/VaD Intercept (ref: AD)	-	-	-	-	-1.09 (0.44)	-1.08 (0.43)
FTD Intercept (ref: AD)	-	-	-	-	-0.17 (0.93)	-0.14 (0.93)
PDD/DLB Intercept (ref: AD)	-	-	-	-	-3.69 (0.72)	-3.66 (0.72)
Other Intercept (ref: AD)	-	-	-	-	-1.50 (1.16)	-1.50 (1.15)
VaD Slope	-	-	-	-	0.50 (0.27)	0.49 (0.27)

(ref: AD)						
Mixed Slope	-	-	-	-	0.03 (0.20)	0.05 (0.20)
(ref: AD)						
FTD Slope	-	-	-	-	-0.47 (0.42)	-0.51 (0.41)
(ref: AD)						
PDD/DLB Slope	-	-	-	-	-0.11 (0.35)	-0.04 (0.36)
(ref: AD)						
Other Slope	-	-	-	-	0.29 (0.54)	0.28 (0.54)
(ref: AD)						

Note: estimate, est; standard error; SE; vascular dementia, VaD; mixed Alzheimer's and vascular, Mixed AD/VaD; frontotemporal dementia, FTD; Parkinson's disease dementia/dementia with Lewy bodies, PDD/DLB; Unspecified dementia/Other, Other; Quality of Life in Alzheimer's Disease, QoL-AD; Satisfaction with life scale, SwLS; World Health Organization-Five Well-being Index, WHO-5.

Supplementary Table S4. Latent growth curve modelling of quality of life over time and the effects of covariates. Measures were adjusted for age, sex, and diagnosis.

Measures	Mean intercept (estimate, 95% CI)	Mean slope (estimate, 95% CI)
Unconditional Model	36.73 (36.43 – 37.03)*	-0.15 (-0.31 – 0.02)
Variance	28.70 (25.12 – 32.28)*	2.58 (1.04 – 4.12)*
Demographics		
Diagnosis Type [a] (ref: AD)		
VaD	-2.70 (-3.69 – -1.71)*	0.76 (0.18 – 1.34)*
Mixed AD/VaD	-1.41 (-2.16 – -0.66)*	-0.00 (-0.41 – 0.40)
FTD	0.09 (-1.50 – 1.68)	-0.54 (-1.39 – 0.31)
PDD/DLB	-4.80 (-6.02 – -3.58)*	-0.62 (-1.37 – 0.14)
Unspecified/Other	-3.00 (-4.97 – -1.02)*	0.18 (-0.94 – 1.30)
Age [a] (years)	0.07 (0.03 – 0.10)*	-0.01 (-0.03 – 0.01)
Sex [a] (Ref: Female)		
Male	-0.29 (-0.91 – 0.32)	-0.13 (-0.46 – 0.20)
Education (Ref: School leaving certificate at age 18)		
No qualifications	-1.25 (-2.01 - -0.49)*	0.06 (-0.37 – 0.48)
School leaving certificate at age 16	0.10 (-0.77- 0.97)	-0.00 (-0.50 – 0.49)
University	0.12 (-0.71 – 0.95)	-0.02 (-0.48 – 0.43)
Living Situation (Ref: Lives with spouse)		
Lives alone	-1.55 (-2.35 - -0.75)*	-0.47 (-0.92 - -0.01)*
Lives with others	-1.20 (-2.51 - 0.10)	-0.23 (-1.01 – 0.55)
Social class (Ref: High I-Professional /II-Managerial and technical)		
Middle (III-NM-Skilled non-manual /III-M-Skilled manual)	-0.17 (-0.80 – 0.48)	-0.06 (-0.42 – 0.30)

Low (IV-Partly skilled /V-Unskilled /armed forces)	-2.02 (-2.95 - -1.10)*	0.34 (-0.17 – 0.85)
Standing in society	1.18 (1.01 – 1.34)*	-0.10 (-0.20 - -0.01)*
Cognition, functional ability and awareness		
ACE-III total	0.03 (0.01 – 0.05)*	-0.01 (-0.02 – 0.01)
ACE-III fluency	0.21 (0.12 – 0.31)*	-0.01 (-0.07 – 0.04)
ACE-III attention	0.08 (-0.02 – 0.18)	-0.06 (-0.12 – 0.00)
ACE-III visuospatial	0.23 (0.14 – 0.32)*	-0.01 (-0.06 – 0.05)
ACE-III memory	-0.07 (-0.12 - -0.01)*	-0.01 (-0.04 – 0.03)
ACE-III language	0.14 (0.06 – 0.23)*	-0.05 (-0.10 – 0.01)
Functional ability	-0.21 (-0.25 - -0.17)*	0.02 (-0.01 – 0.04)
Low Awareness	4.15 (2.86 – 5.44)*	-0.62 (-1.42 – 0.17)
Physical health		
CCI score	-0.75 (-0.90 - -0.59)*	0.10 (0.01 – 0.18)*
Self-rated health	2.62 (2.39 – 2.85)*	-0.36 (-0.50 - -0.21)*
Social contact and engagement		
Social isolation	0.27 (0.22 – 0.31)*	-0.02 (-0.05 - 0.01)
Cultural capital	0.28 (0.23 – 0.33)*	-0.03 (-0.06 – 0.00)
Psychological measures		
Depression	-1.73 (-1.83 - -1.62)*	0.25 (0.17 – 0.33)*
Loneliness	-1.65 (-1.83 - -1.47)*	0.15 (0.03 – 0.26)*
Self-esteem	0.78 (0.71 – 0.85)*	-0.08 (-0.13 - -0.04)*
Optimism	0.76 (0.68 – 0.84)*	-0.08 (-0.12 - -0.03)*

Note. confidence intervals, CI; Alzheimer’s disease, AD; vascular dementia, VaD; Mixed Alzheimer’s and vascular, Mixed AD/VaD; Frontotemporal dementia, FTD; Parkinson’s disease dementia/dementia with Lewy bodies, PDD/DLB; Addenbrooke’s Cognitive Examination-III, ACE-III; Charlson Comorbidity Index, CCI.

[a] unadjusted * 95% confidence intervals do not span 1.

Supplementary Table S5. Latent growth curve modelling of 'living well' over time and the effects of covariates. Measures were adjusted for age, sex, and diagnosis.

**Measures**	**Mean intercept (estimate, 95% CI)**	**Mean slope (estimate, 95% CI)**
Unconditional Model	36.71 (36.42 – 37.00)*	-0.17 (-0.31 – 0.03)
Variance	24.05 (20.96 – 37.14)*	2.25 (1.08 – 3.43)*
Demographics		
Diagnosis Type [a] (ref: AD)		
VaD	-2.60 (-3.50 – -1.70)*	0.57 (0.08 – 1.05)*
Mixed AD/VaD	-1.34 (-2.02 – -0.65)*	0.11 (-0.24 – 0.46)
FTD	-0.44 (-1.87 – 1.00)	-0.54 (-1.29 – 0.21)
PDD/DLB	-4.82 (-5.92 – -3.72)*	-0.35 (-0.98 – 0.28)
Unspecified/Other	-2.23 (-3.99 – -0.47)*	0.11 (-0.83 – 1.05)
Age [a] (years)	0.08 (0.05 – 0.12)*	-0.01 (-0.02 – 0.01)
Sex [a] (Ref: Female)		
Male	0.03 (-0.53 – 0.59)	-0.06 (-0.34 – 0.22)
Education (Ref: School leaving certificate at age 18)		
No qualifications	-0.96 (-1.65 - -0.26)*	0.14 (-0.22 – 0.51)
School leaving certificate at age 16	-0.06 (-0.73 – 0.84)	-0.02 (-0.43 – 0.40)
University	-0.02 (-0.77 – 0.72)	0.09 (-0.30 – 0.47)
Living Situation (Ref: Lives with spouse)		
Lives alone	-2.00 (-2.72 - -1.28)*	-0.23 (-0.62 – 0.16)
Lives with others	-0.73 (-1.90 – 0.45)	-0.27 (-0.94 – 0.40)

Social class (Ref: High I-Professional /II-Managerial and technical)		
Middle (III-NM-Skilled non-manual /III-M-Skilled manual)	0.05 (-0.63 – 0.53)	-0.12 (-0.42 – 0.19)
Low (IV-Partly skilled /V-Unskilled /armed forces)	-1.50 (-2.33 - -0.67)*	0.20 (-0.25 – 0.64)
Standing in society	1.07 (0.91 – 1.22)*	-0.06 (-0.14 – 0.03)
Cognition, functional ability and awareness		
ACE-III total	0.01 (-0.01 – 0.03)	0.00 (-0.01 – 0.01)
ACE-III fluency	0.12 (0.04 – 0.21)*	0.01 (-0.04 – 0.06)
ACE-III attention	0.00 (-0.09 – 0.09)	-0.01 (-0.06 – 0.04)
ACE-III visuospatial	0.17 (0.09 – 0.25)*	0.03 (-0.02 – 0.08)
ACE-III memory	-0.10 (-0.15 – -0.05)*	0.00 (-0.02 – 0.03)*
ACE-III language	0.09 (0.02 – 0.17)*	0.00 (-0.04 – 0.04)
Functional ability	-0.18 (-0.21 - -0.14)*	0.01 (-0.01 – 0.03)*
Low Awareness	3.51 (2.33 – 4.69)*	-0.55 (-1.25 – 0.16)
Physical health		
CCI score	-0.67 (-0.81 - -0.52)*	0.06 (-0.02 – 0.13)
Self-rated health	2.43 (2.21 – 2.65)*	-0.23 (-0.36 – -0.11)*
Social contact and engagement		
Social isolation	0.24 (0.19 – 0.28)*	0.24 (0.19 – 0.29)*
Cultural capital	-0.02 (-0.05 - -0.00)	-0.02 (-0.04 – 0.01)
Psychological measures		
Depression	-1.70 (-1.79 - -1.60)*	0.22 (0.15 – 0.28)*
Loneliness	-1.64 (-1.81 - -1.47)*	0.14 (0.04 – 0.24)*
Self-esteem	0.75 (0.69 – 0.82)*	-0.07 (-0.10 - -0.03)*
Optimism	0.73 (0.66 – 0.80)*	-0.06 (-0.10 - -0.01)*

Note. estimate, est; confidence intervals, CI; Alzheimer's disease, AD; vascular dementia, VaD; mixed Alzheimer's and vascular, Mixed AD/VaD; frontotemporal dementia, FTD; Parkinson's disease dementia/dementia with Lewy bodies, PDD/DLB; Addenbrooke's Cognitive Examination-III, ACE-III; Charlson Comorbidity Index, CCI.

[a] unadjusted

* 95% confidence intervals do not span 1.

Supplementary Table S6. Testing increasing numbers of classes in LCGA, GMM-CI and GMM-CV models for quality of life. In the LCGA, variances and covariances are set to zero. In a GMM-CI, variances and covariances are freed between classes but not within classes (variances fixed across classes). In a GMM-CV all parameters are freed.

**Fit statistics**	**1 class**	**2 class**	**3 class**	**4 class**	**5 class**
LCGA					
LL	-10023.5	-9652.1	-9494.7	-9416.4	-9398.2
BIC	20083.5	19362.7	19069.8	18935.1	18920.7
ssaBIC	20067.6	19337.3	19034.8	18890.6	18866.7
Entropy	1	0.679	0.691	0.704	0.682
adj LMR-LRT	-	710.29	301.11	149.75	34.78
(p)		(0.000)	(0.006)	(0.001)	(0.007)
BLRT	-	742.68	314.85	156.58	36.37
(p)		(0.000)	(0.000)	(0.000)	(0.000) [a]
Group size (%)					
C1	100	66.6	23.4	29.4	13.2
C2	-	33.4	57.8	11.5	4.6
C3	-	-	18.8	10.0	10.0
C4	-	-	-	49.1	42.5
C5	-	-	-	-	29.6
GMM-CI					
LL	-9420.4	-9395.2	-9386.7	-9379.1	-
BIC	18899.2	18870.7	18875.8	18882.3	-
ssaBIC	18873.8	18835.8	18831.3	18828.3	-
Entropy	1	0.652	0.719	0.659	-

adj LMR-LRT	-	48.20	16.11	14.70	-
(p)		(0.009)	(0.018)	(0.014)	
BLRT	-	50.39	16.84	15.37	-
(p)		(0.000)	(0.000) [a]	(0.000)	
Group size (%)					-
C1	100	82.2	3.4	74.9	
C2	-	17.8	81.0	13.7	-
C3	-	-	15.6	7.5	-
C4	-	-	-	3.8	-
C5	-	-	-	-	-
GMM-CV					
LL	-9420.4	-9388.31	-	-	-
BIC	18899.2	18871.62	-	-	-
ssaBIC	18873.8	18830.32	-	-	-
Entropy	1	0.730	-	-	-
adj LMR-LRT	-	62.39	-	-	-
(p)		(0.000)			
BLRT	-	64.09	-	-	-
(p)		(0.000)			
Group size (%)			-	-	-
C1	100	14.8			
C2	-	85.2	-	-	-
C3	-	-	-	-	-
C4	-	-	-	-	-
C5	-	-	-	-	-

Note. latent class growth analysis, LCGA; growth mixture modelling – class invariant, GMM-CI; growth mixture modelling – class varying, GMM-CV; log-likelihood, LL; Bayesian Information Criterion, BIC; samples size adjusted BIC; ssaBIC; Lo-Mendell-Rubin adjusted Likelihood Ratio Test, adj LMR-LRT; Bootstrap Likelihood Ratio Test, BLRT; class 1-class 5, C1-C5. Where no solution is presented, the model had convergence difficulties.

[a] P value may not be reliable and adj LMR-LRT should be used for model comparison.

Supplementary Table S7. Average latent class probabilities for most likely latent class membership (row) by latent class (column) for the 4-class model of quality of life.

**Class**	**Class 1. Stable**	**Class 2. Stable Lower**	**Class 3. Declining**	**Class 4. Improving**
**Class 1. Stable**	0.719	0.196	0.085	0.000
**Class 2. Stable Lower**	0.060	0.663	0.061	0.016
**Class 3. Declining**	0.072	0.171	0.662	0.096
**Class 4. Improving**	0.001	0.159	0.145	0.695

Note. Each column represents classification probabilities (averages of the individual probabilities in each class) for the 4-class solution of the estimated GMM-CI, and each row represents the classification probabilities for the most likely class. If classes were perfectly separated, the diagonal components would be 1, and the off diagonals would be 0.

Supplementary Table S8. Testing increasing numbers of classes in LCGA, GMM-CI and GMM-CV models for 'living well'. In the LCGA, variances and covariances are set to zero. In a GMM-CI, variances and covariances are freed between classes but not within classes (variances fixed across classes). In a GMM-CV all parameters are freed.

**Fit statistics**	**1 class**	**2 class**	**3 class**	**4 class**	**5 class**
LCGA					
LL	-33736.2	-33123.2	-33001.0	-32960.9	-32937.1
BIC	67626.6	66422.5	66200.6	66142.0	66116.3
ssaBIC	67559.9	66346.3	66114.8	66046.7	66011.5
Entropy	1	0.802	0.660	0.664	0.653
adj LMR-LRT (p)	-	1172.8 (0.000)	233.4 (0.002)	77.1 (0.027)	45.7 (0.149)
BLRT (p)	-	1226.1 (0.000)	245.0 (0.000)	80.6 (0.000)	47.7 (0.000)
Group size (%)					
C1	100	73.9	45.2	52.0	50.6
C2	-	26.1	40.0	9.8	8.5
C3	-	-	14.8	23.4	17.8
C4	-	-	-	14.8	8.7
C5	-	-	-	-	14.5
GMM-CI					
LL	-33000.7	-32941.0	-32925.1	-32906.3	-
BIC	66177.5	66080.2	66070.5	66054.7	-
SSABIC	66101.3	65994.4	65975.2	65949.9	-
Entropy	1	0.732	0.697	0.690	-

adj LMR-LRT (p)	-	114.1 (0.006)	30.4 (0.303)	36.1 (0.004)	-
BLRT (p)	-	119.3 (0.000)	31.7 (0.000)	37.8 (0.000)	-
Group size (%)					-
C1	100	80.6	75.5	72.0	
C2	-	19.4	15.3	12.7	-
C3	-	-	9.2	9.9	-
C4	-	-	-	5.5	-
C5	-	-	-	-	-
GMM-CV					
LL	-33000.7	-32926.9	-	-	-
BIC	66177.5	66066.7	-	-	-
SSABIC	66101.3	65974.5	-	-	-
Entropy	1	0.634	-	-	-
adj LMR-LRT (p)	-	143.6 (0.017)	-	-	-
BLRT (p)	-	147.5 (0.000) [a]	-	-	-
Group size (%)			-	-	-
C1	100	25.6			
C2	-	74.4	-	-	-
C3	-	-	-	-	-
C4	-	-	-	-	-
C5	-	-	-	-	-

Note. latent class growth analysis, LCGA; growth mixture modelling – class invariant, GMM-CI; growth mixture modelling – class varying, GMM-CV; log-likelihood, LL;

Bayesian Information Criterion, BIC; samples size adjusted BIC; ssaBIC; Lo-Mendell-Rubin adjusted Likelihood Ratio Test, adj LMR-LRT; Bootstrap Likelihood Ratio Test, BLRT; class 1-class 5, C1-C5. Where no solution is presented, the model had convergence difficulties.

[a] P value may not be reliable and adj LMR-LRT should be used for model comparison.

Supplementary Table S9. Average latent class probabilities for most likely latent class membership (row) by latent class (column) for the 4-class model of 'living well'.

**Class**	**Class 1. Stable**	**Class 2. Stable Lower**	**Class 3. Declining**	**Class 4. Improving**
**Class 1. Stable**	0.879	0.033	0.070	0.018
**Class 2. Stable Lower**	0.112	0.679	0.068	0.141
**Class 3. Declining**	0.181	0.094	0.724	0.001
**Class 4. Improving**	0.126	0.151	0.002	0.721

Note. Each column represents classification probabilities (averages of the individual probabilities in each class) for the 4-class solution of the estimated GMM-CI, and each row represents the classification probabilities for the most likely class. If classes were perfectly separated, the diagonal components would be 1, and the off diagonals would be 0.

Supplementary Table S10. Characteristics of each latent 'living well' class.

Misclassification error derived from the posterior probabilities is taken into account.

**Measures**	**Class 1. Stable** [a]	**Class 2. Stable Lower** [b]	**Class 3. Declining** [c]	**Class 4. Improving** [d]
Living Well measures	(mean (sd))	(mean (sd))	(mean (sd))	(mean (sd))
QoL-AD				
T1	38.7 (4.7)	29.0 (4.6)	36.9 (4.6)	28.6 (4.8)
T2	38.9 (4.7)	29.0 (4.9)	33.9 (5.4)	33.4 (5.0)
T3	38.7 (4.4)	29.1 (4.2)	30.9 (4.6)	36.9 (5.1)
SwLS				
T1	28.0 (4.5)	18.4 (5.8)	26.1 (5.4)	18.7 (5.8)
T2	28.1 (4.5)	18.7 (6.8)	23.2 (6.5)	23.2 (6.4)
T3	28.4 (4.4)	17.8 (6.4)	19.1 (6.4)	26.0 (5.4)
WHO-5				
T1	67.6 (15.7)	33.9 (17.5)	62.3 (16.7)	33.7 (16.5)
T2	67.4 (16.7)	34.6 (17.8)	49.2 (19.1)	49.2 (17.9)
T3	68.2 (16.4)	34.0 (16.8)	38.2 (17.5)	60.0 (16.1)
Demographic variables	(n, (%))	(n, (%))	(n, (%))	(n, (%))
Diagnosis Type				
AD	652 (58.8%)	78 (39.8%)	80 (52.4%)	35 (41.8%)
VaD	107 (9.6%)	28 (14.3%)	15 (10.2%)	15 (17.4%)
Mixed AD/VaD	235 (21.2%)	45 (23.1%)	30 (20.0%)	19 (22.5%)
FTD	41 (3.7%)	7 (3.4%)	7 (4.8%)	3 (3.6%)
PDD/DLB	49 (4.4%)	29 (15.1%)	16 (10.7%)	10 (12.7%)

Unspecified/Other	25 (2.3%)	8 (4.2%)	3 (2.0%)	2 (2.1%)
Age				
<65	83 (7.5%)	27 (14.0%)	15 (9.6%)	11 (13.5%)
65-69	118 (10.7%)	28 (14.2%)	18 (11.5%)	13 (15.9%)
70-74	182 (16.4%)	37 (19.1%)	26 (17.3%)	13 (15.0%)
75-79	265 (23.9%)	42 (21.4%)	39 (25.5%)	20 (23.1%)
80+	461 (41.6%)	61 (31.2%)	55 (36.0%)	27 (32.5%)
Mean age	76.9 (8.1)	74.3 (10.0)	76.1 (8.4)	74.7 (9.8)
Sex				
Male	742 (66.9%)	128 (65.8%)	101 (66.2%)	58 (68.5%)
Female	366 (33.1%)	67 (34.2%)	51 (33.8%)	27 (31.5%)
Education				
No qualifications	295 (27.0%)	60 (31.0%)	42 (27.9%)	29 (35.1%)
School leaving certificate at age 16	199 (18.1%)	33 (17.0%)	26 (17.4%)	13 (15.5%)
School leaving certificate at age 18	378 (34.5%)	61 (31.5%)	52 (34.2%)	25 (30.2%)
University	223 (20.4%)	40 (20.6%)	31 (20.5%)	16 (19.2%)
Living Situation				
Lives alone	195 (17.7%)	48 (24.5%)	30 (19.7%)	16 (18.6%)
Lives with spouse	845 (76.8%)	136 (69.9%)	112 (74.1%)	63 (74.9%)
Lives with others	59 (5.4%)	11 (5.6%)	9 (6.1%)	5 (6.5%)
Social Class				
Low (IV-Partly skilled /V-Unskilled /armed forces)	135 (12.9%)	36 (19.6%)	19 (13.3%)	15 (18.3%)
Middle (III-NM-Skilled non-manual /III-M-Skilled manual)	431 (41.2%)	73 (39.7%)	65 (45.5%)	31 (37.8%)

High (I-Professional /II-Managerial and technical)	479 (45.8%)	75 (40.8%)	59 (41.3%)	36 (43.9%)
	(mean (sd))	(mean (sd))	(mean (sd))	(mean (sd))
Standing in Society	6.9 (1.6)	5.8 (1.8)	6.5 (1.6)	6.2 (1.8)
Cognition, functional impairment and awareness				
ACE-III total	69.3 (13.0)	70.3 (13.2)	67.7 (13.8)	68.1 (13.1)
ACE-III fluency	6.9 (3.0)	6.7 (3.1)	6.3 (3.1)	6.3 (3.1)
ACE-III attention	13.9 (2.9)	14.2 (2.9)	13.7 (3.2)	13.4 (3.1)
ACE-III visuospatial	12.7 (3.1)	12.0 (3.6)	12.0 (3.6)	11.9 (3.5)
ACE-III memory	13.2 (5.4)	15.0 (5.2)	13.5 (5.5)	14.1 (5.1)
ACE-III language	22.5 (3.6)	22.5 (3.6)	22.3 (3.7)	22.2 (3.8)
Functional ability	8.7 (7.3)	12.7 (8.0)	10.7 (7.9)	12.9 (8.2)
Awareness (n, %)	(n, (%))	(n, (%))	(n, (%))	(n, (%))
Low	71 (6.9%)	3 (1.7%)	8 (5.8%)	1 (1.9%)
Rest of cohort	949 (93.1%)	178 (98.3%)	130 (94.2%)	77 (98.1%)
Physical health	(mean (sd))	(mean (sd))	(mean (sd))	(mean (sd))
CCI symptoms	6.9 (2.1)	7.5 (2.5)	7.0 (2.2)	7.3 (2.3)
Self-Rated Health	4.0 (1.1)	2.9 (1.0)	3.7 (1.1)	3.1 (1.1)
Social contact and engagement				
Social Isolation	15.7 (6.0)	12.6 (5.7)	15.0 (6.7)	12.5 (6.0)
Cultural Capital	23.3 (5.6)	21.3 (5.4)	22.5 (5.5)	21.1 (5.1)
Psychological measures				
Depression	2.0 (1.8)	5.4 (2.4)	2.7 (2.0)	5.3 (2.4)

Loneliness	1.1 (1.2)	2.6 (1.9)	1.4 (1.4)	2.4 (1.9)
Self-Esteem	30.2 (3.5)	26.3 (3.7)	29.1 (3.2)	27.0 (4.2)
Optimism	15.6 (3.2)	12.3 (3.7)	14.7 (3.3)	13.1 (3.9)

Note. Alzheimer's disease, AD; vascular dementia, VaD; frontotemporal dementia, FTD; Parkinson's disease dementia, PDD; dementia with Lewy bodies, DLB; Addenbrooke's Cognitive Examination-III, ACE-III; Charlson Comorbidity Index, CCI.

[a] N = 1108, 72.0%

[b] N = 195, 12.7%

[c] N = 153, 9.9%

[d] N = 85, 5.5%

[e] Missing includes those that are unclassifiable and those that selected not applicable

Supplementary Table S11. Predicting class membership for 'living well' using multinomial logistic regression, adjusting for sex, age and diagnosis. Reference class is 'Stable'. Class membership error is accounted for.

a) Univariable models

**Measures**	**Class 1. Stable** OR (95% CI)	**Class 2. Stable Lower** OR (95% CI)	**Class 3. Declining** OR (95% CI)	**Class 4. Improving** OR (95% CI)
Demographics				
Diagnosis Type [a] (ref: AD)				
VaD	ref	3.24 (1.55 – 6.77)*	1.10 (0.28 – 4.36)	2.77 (0.74 – 10.37)
Mixed AD/VaD	ref	1.80 (0.88 – 3.68)	1.18 (0.46 – 3.04)	2.48 (0.86 – 7.13)
FTD	ref	1.01 (0.14 – 7.35)	1.37 (0.24 – 7.96)	2.10 (0.28 – 15.75)
PDD/DLB	ref	14.08 (5.73 – 34.60)*	6.97 (1.81 – 26.82)*	8.19 (1.69 – 39.65)*
Unspecified/Other	ref	4.35 (1.61 – 11.74)*	0.31 (0.01 – 80.68)	0.00 (0.00 – 0.00)
Age [a] (years)	ref	0.95 (0.91 – 1.01)	0.98 (0.93 – 1.02)	0.94 (0.86 – 1.04)
Sex [a] (ref: Female)				
Male	ref	1.09 (0.66 – 1.82)	1.19 (0.56 – 2.53)	0.98 (0.36 – 2.51)

Education (ref: School leaving certificate at age 18)				
No qualifications	ref	1.55 (0.71 – 3.39)	1.41 (0.50 – 4.00)	3.98 (1.25 – 12.66)*
School leaving certificate at age 16	ref	1.17 (0.52 – 2.61)	0.90 (0.31 – 2.64)	0.27 (0.04 – 1.69)
University	ref	1.54 (0.72 – 3.27)	1.04 (0.35 – 3.09)	0.77 (0.10 – 6.03)
Living Situation (ref: Spouse)				
Lives alone	ref	3.30 (1.75 – 6.21)*	1.33 (0.45 – 3.96)	0.62 (0.08 – 5.03)
Lives with others	ref	1.44 (0.30 – 6.99)	2.09 (0.49 – 9.03)	2.29 (0.38 – 13.91)
Social Class (ref: (I-Professional /II-Managerial and technical))	ref			
Low (IV-Partly skilled /V-Unskilled /armed forces)	ref	2.15 (1.06 – 4.35)*	1.02 (0.14 – 7.49)	2.28 (0.70 – 7.43)
Middle (III-NM-Skilled non-manual /III-M-Skilled manual)		0.86 (0.40 – 1.84)	1.99 (0.85 – 4.64)	1.16 (0.33 – 4.05)
Standing in Society	ref	0.39 (0.25 – 0.58)*	0.84 (0.66 – 1.08)	0.96 (0.76 – 1.21)
Cognition, functional ability and awareness				
ACE-III total	ref	1.01 (0.98 – 1.05)	0.98 (0.95 – 1.02)	0.98 (0.95 – 1.01)
ACE-III fluency	ref	1.03 (0.85 -1.25)	0.86 (0.74 – 0.99)*	0.85 (0.63 – 1.14)
ACE-III attention	ref	1.11 (0.99 – 1.24)	0.91 (0.75 – 1.10)	0.86 (0.73 – 1.02)

ACE-III visuospatial	ref	0.90 (0.83 – 0.98)*	0.93 (0.82 – 1.05)	0.98 (0.84 – 1.15)
ACE-III memory	ref	1.09 (1.03 – 1.15)*	1.01 (0.92 – 1.11)	0.99 (0.92 – 1.07)
ACE-III language	ref	0.97 (0.89 – 1.06)	1.00 (0.89 – 1.14)	0.97 (0.87 – 1.07)
Functional ability	ref	1.09 (1.05 – 1.12)*	1.06 (1.00 – 1.13)*	1.12 (1.04 – 1.20)*
Low Awareness	ref	0.17 (0.03 – 0.95)*	0.83 (0.12 – 5.72)	0.16 (0.01 – 3.12)
Physical health				
CCI symptoms	ref	1.52 (1.26 – 1.83)*	1.14 (0.89 – 1.44)	1.46 (1.19 – 1.78)*
Self-Rated Health	ref	0.17 (0.11 – 0.27)*	0.72 (0.46 – 1.14)	0.32 (0.17 – 0.62)*
Social contact and engagement				
Social Isolation	ref	0.86 (0.82 – 0.90)*	1.00 (0.90 – 1.11)	0.86 (0.80 – 0.92)*
Cultural Capital	ref	0.87 (0.81 – 0.94)*	0.95 (0.87 – 1.03)	0.84 (0.77 – 0.91)*
Psychological measures				
Depression	ref	3.72 (2.87 – 4.82)*	1.40 (1.11 – 1.76)*	3.55 (2.56 – 4.94)*
Loneliness	ref	2.90 (2.24 – 3.77)*	1.48 (1.07 – 2.06)*	2.71 (1.83 – 4.01)*
Self-Esteem	ref	0.54 (0.46 – 0.63)*	0.78 (0.66 – 0.92)*	0.55 (0.39 – 0.79)*
Optimism	ref	0.60 (0.53 – 0.68)*	0.81 (0.69 – 0.96)*	0.74 (0.63 – 0.88)*

b) Multivariable model

**Measures**	**Class 1. Stable**<br>**OR (95% CI)**	**Class 2. Stable Lower**<br>**OR (95% CI)**	**Class 3. Declining**<br>**OR (95% CI)**	**Class 4. Improving**<br>**OR (95% CI)**
Depression	ref	2.89 (1.93 – 4.32)*	1.10 (0.80 – 1.52)	3.31 (1.26 – 8.73)*
Loneliness	ref	1.86 (1.26 – 2.74)*	1.21 (0.86 – 1.68)	1.57 (0.78 – 3.15)
Self-Esteem	ref	0.81 (0.66 – 1.01)	0.83 (0.68 – 1.02)	0.82 (0.60 – 1.13)
Optimism	ref	0.84 (0.69 – 1.03)	0.90 (0.75 – 1.08)	1.18 (0.59 – 2.35)
Functional ability	ref	0.98 (0.89 – 1.08)	1.04 (0.98 – 1.11)	1.04 (0.95 – 1.19)

Note. Alzheimer's disease, AD; vascular dementia, VaD; frontotemporal dementia, FTD; Parkinson's disease dementia, PDD; dementia with Lewy bodies, DLB; Addenbrooke's Cognitive Examination-III, ACE-III; Charlson Comorbidity Index, CCI; odds ratio, OR; confidence intervals, CI; reference category/class, ref.

[a] unadjusted

* 95% confidence intervals do not span 1

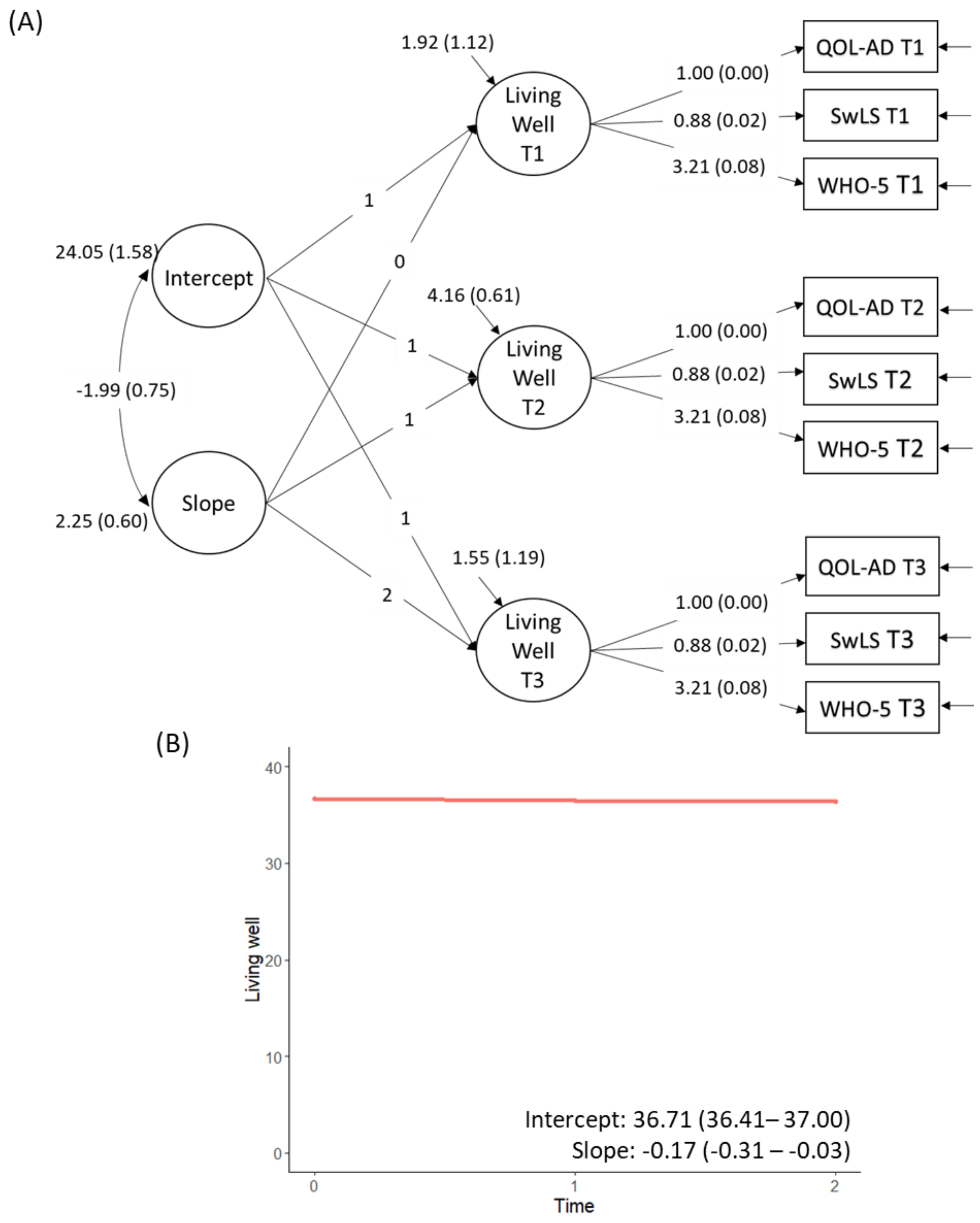

Supplementary Figure S1. (A) The latent growth curve model involves the measurement model where the factors Living Well T1 – Living Well T3 are determined from measures of QoL-AD, SwLS and WHO-5 at T1-T3. QoL-AD is used as the marker variable and loading

fixed to 1. Intercept and slope latent factors are modelled from Living Well, with intercepts fixed to 1, and the occasions of the slopes fixed to 0, 1 and 2. **(B)** The intercept and trajectory of ‘living well’ derived from the model in (A).

(A) Demographic variables

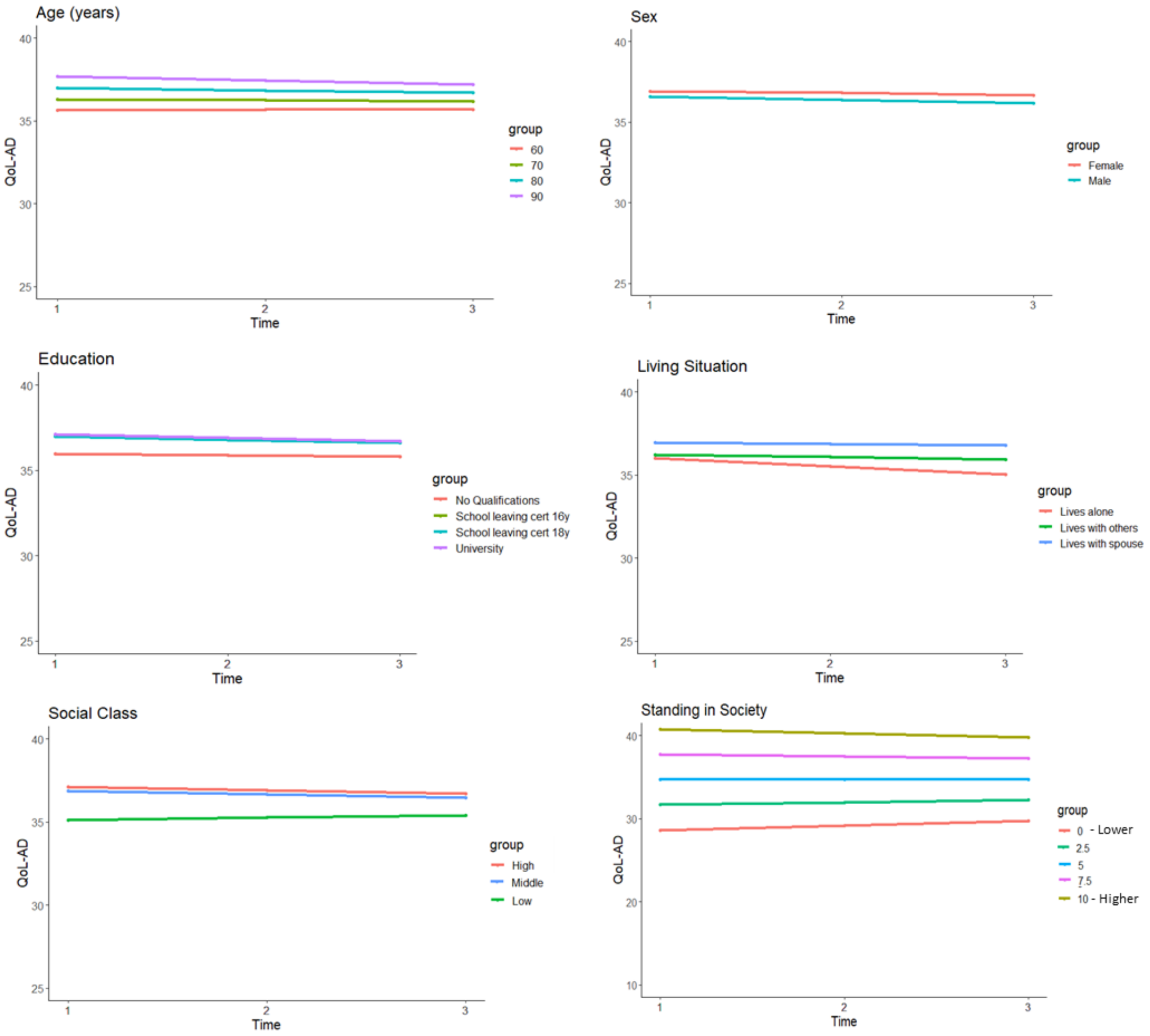

(B) Cognition, functional impairment and awareness

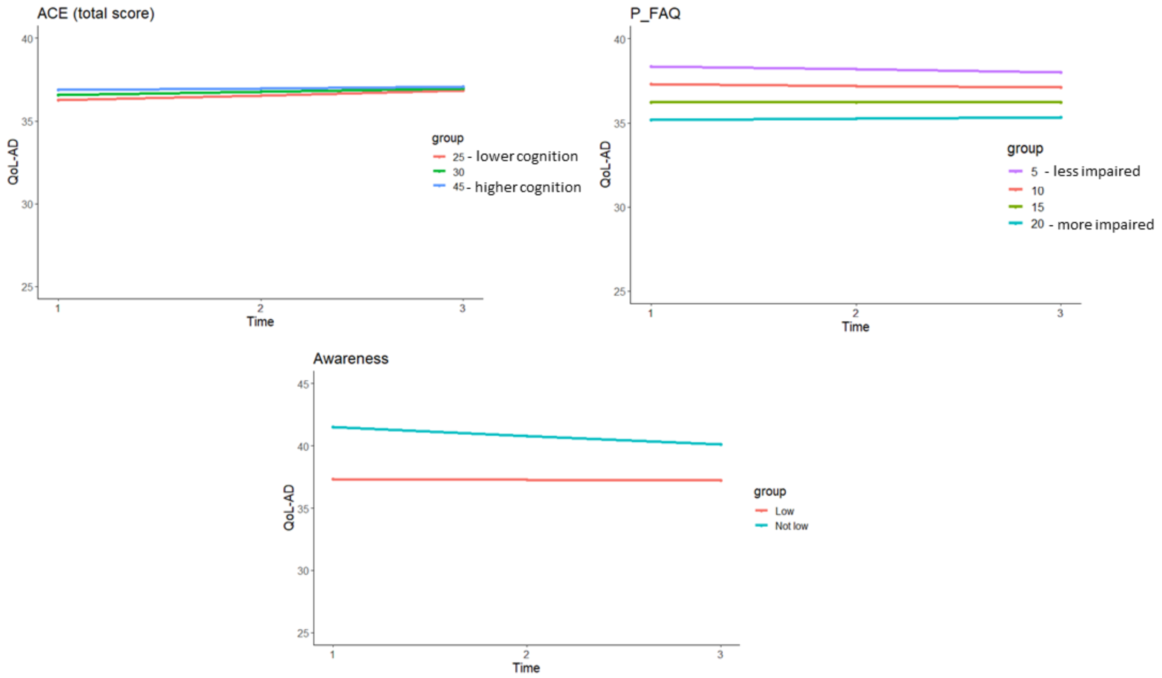

(C) Physical health

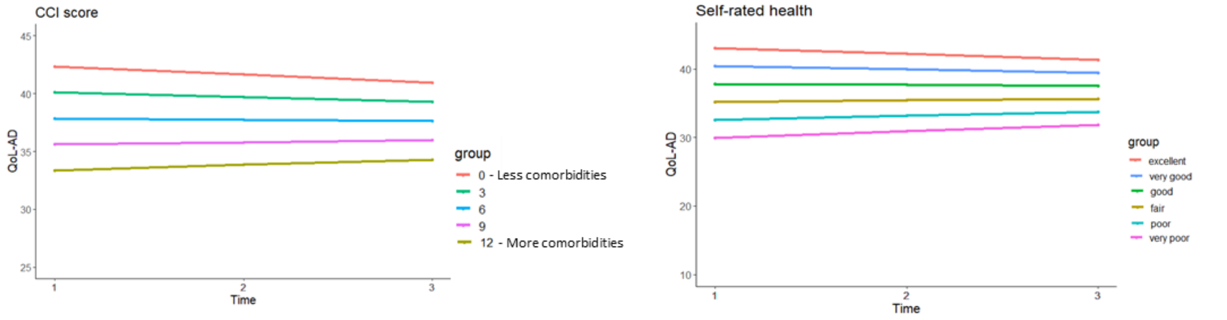

(D) Social contact and engagement

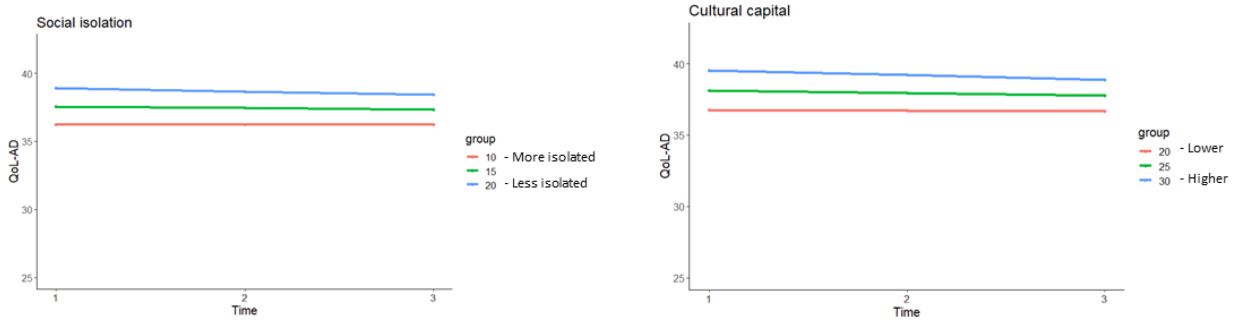

(E) Psychological measures

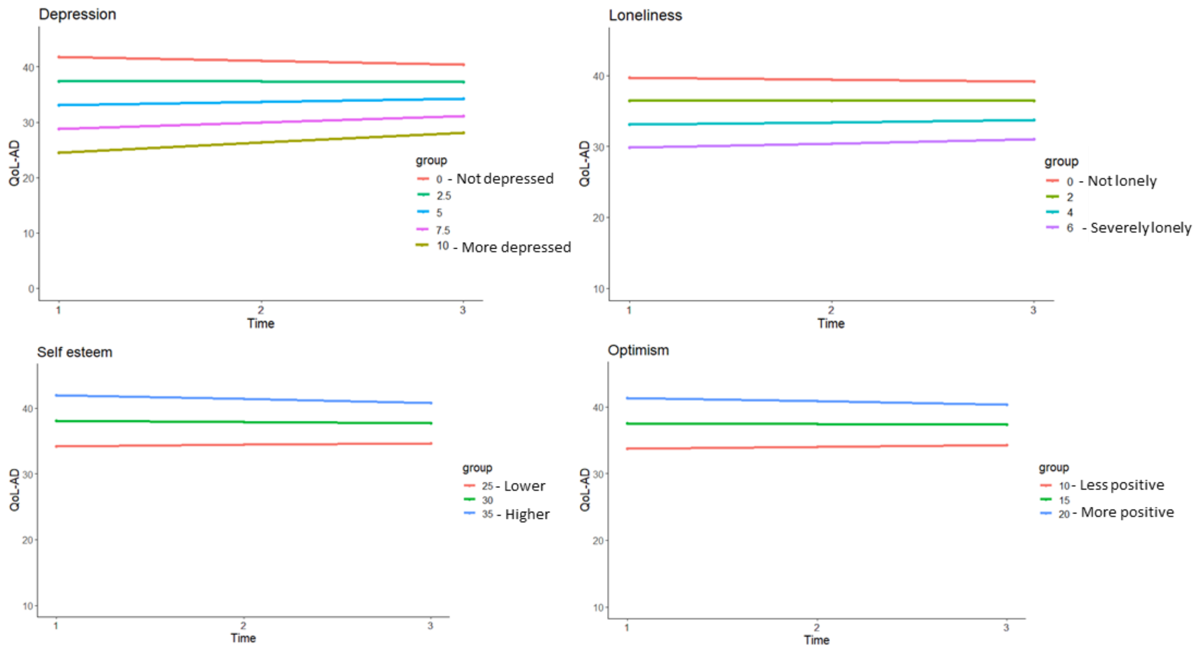

Supplementary Figure S2. Visualisation of the mean intercepts and slopes for Quality of Life by (A) demographic variables, (B) cognition, functional impairment and awareness, (C) physical health, (D) social contact ad engagement and (E) psychological measures. Graphs represent a female, who is 75 years old with Alzheimer's disease. *Note*. For education, all lines fall on top of each other except for 'No Qualifications'.

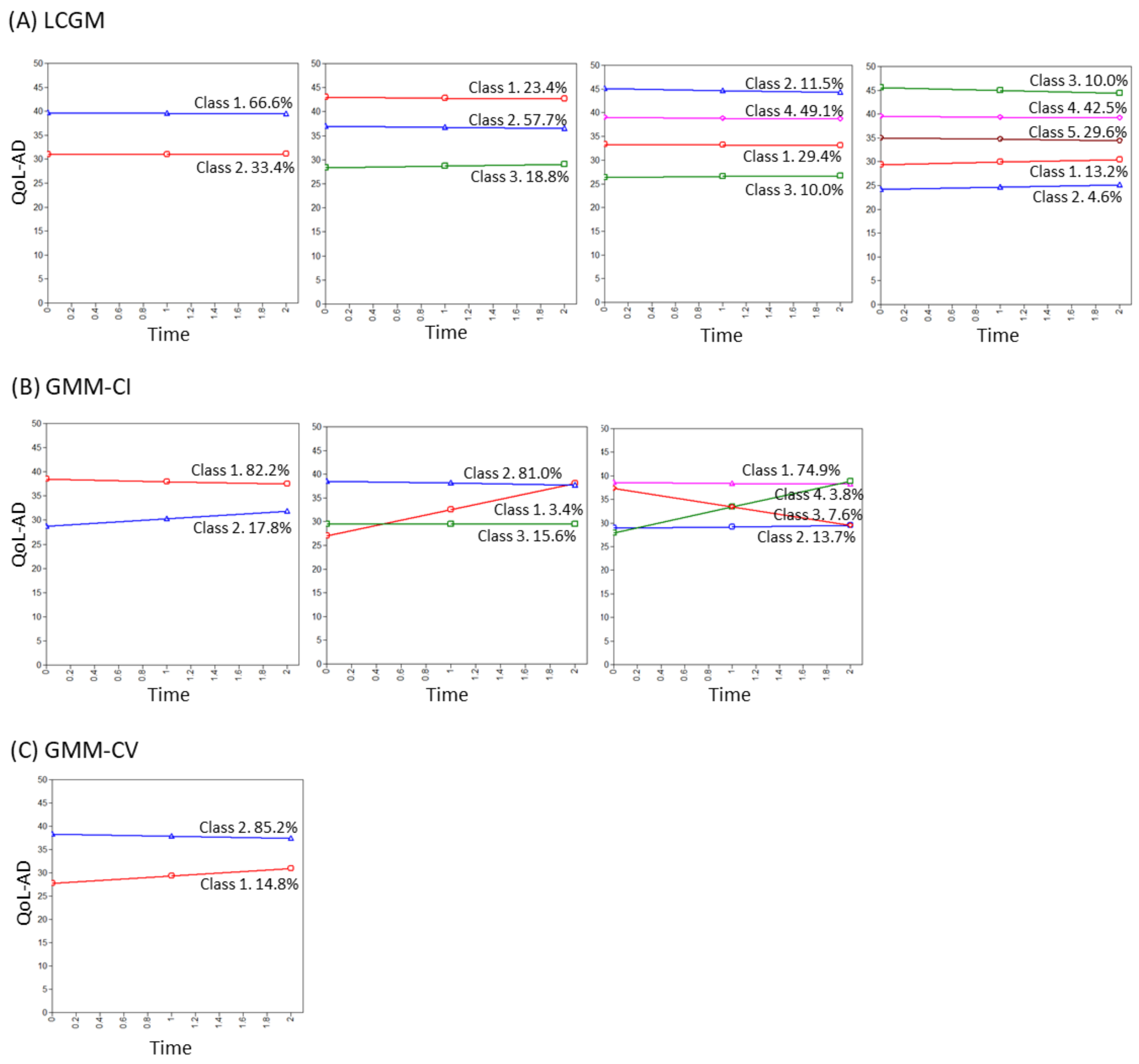

Supplementary Figure S3. Plots of the LCGA, GMM-CI and GMM-CV models for quality of life associated with the models presented in Supplementary Table S6.

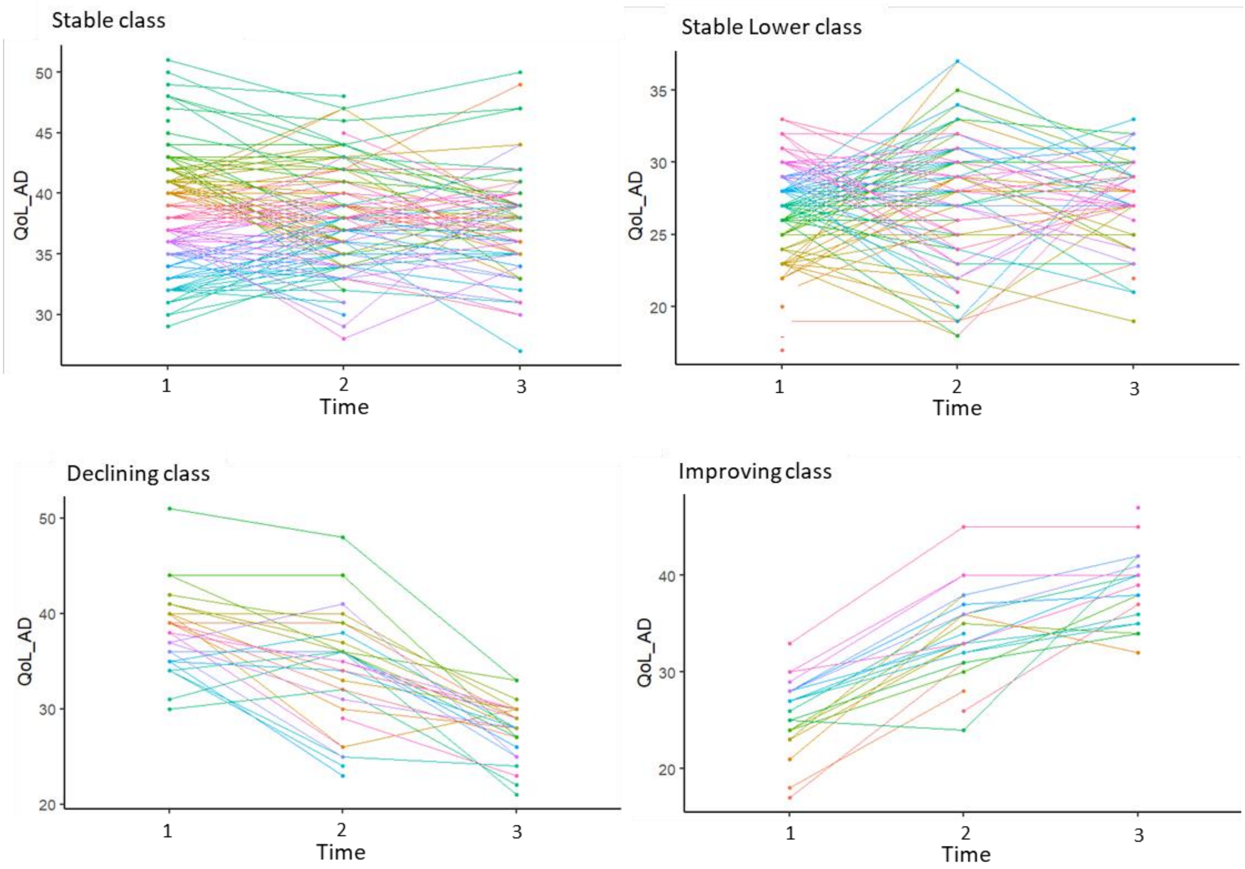

Supplementary Figure S4. Plots of individuals within the four classes of trajectories of Quality of Life. The most likely class is allocated based on posterior probabilities for graphing purposes. For the stable lower and stable classes, random samples of 200 people were selected.

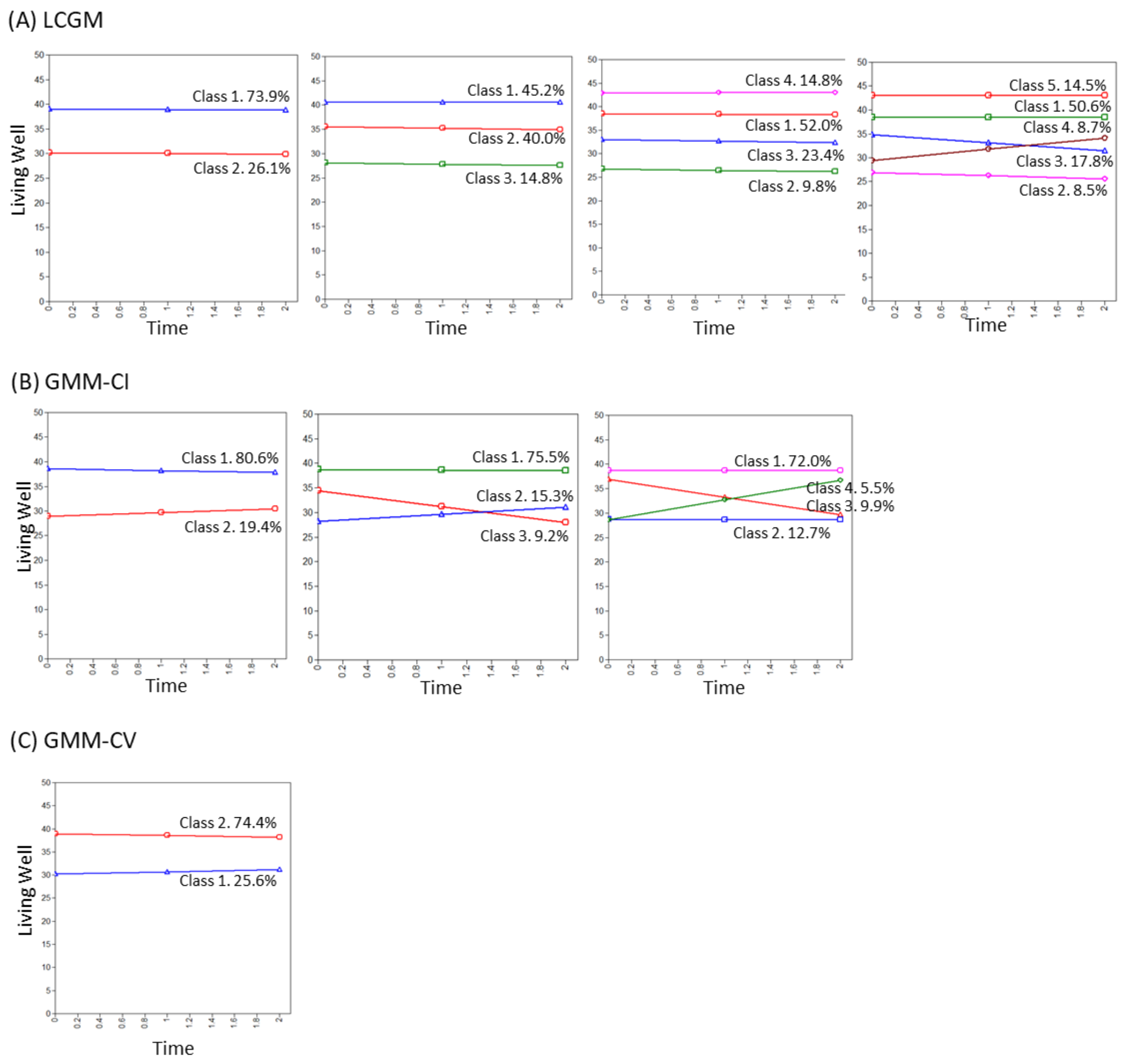

Supplementary Figure S5. Plots of the LCGA, GMM-CI and GMM-CV models for 'living well' associated with the models presented in Supplementary Table S8.

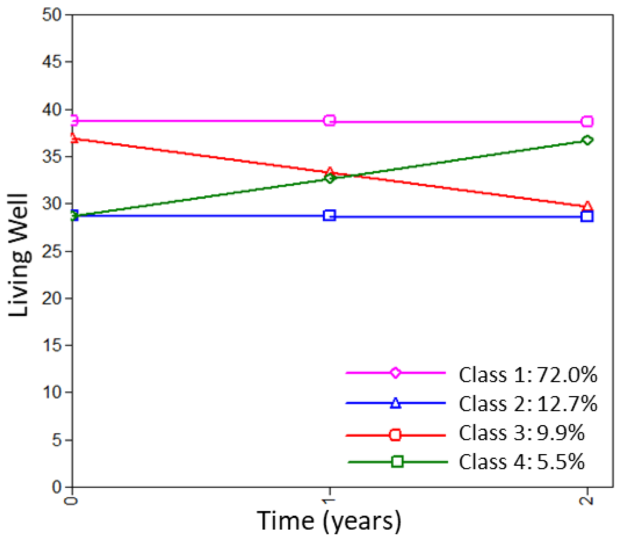

	Class 1. Stable	Class 2. Stable Lower	Class 3. Declining	Class 4. Improving
Intercept	38.69 (38.32 – 39.05)*	28.70 (27.43 – 29.98)*	36.88 (34.72 – 39.04)*	28.65 (26.53 – 30.78)*
Slope	-0.03 (-0.26 – 0.21)	-0.06 (-1.02 – 0.90)	-3.61 (-4.49 – -2.73)*	4.00 (2.66 – 5.35)*
Variance-covariance				
Intercept	9.57 (6.91 – 12.23)	9.57 (6.91 – 12.23)	9.57 (6.91 – 12.23)	9.57 (6.91 – 12.23)
Slope	0.73 (-0.46 – 1.92)	0.73 (-0.46 – 1.92)	0.73 (-0.46 – 1.92)	0.73 (-0.46 – 1.92)
Intercept-slope	-0.82 (-2.25 – 0.62)	-0.82 (-2.25 – 0.62)	-0.82 (-2.25 – 0.62)	-0.82 (-2.25 – 0.62)

Supplementary Figure S6. Trajectories of the 4 classes of ‘living well’ determined from the GMM-CI model: Class 1 Stable, Class 2 Stable Lower, Class 3 Declining, Class 4 Improving. The mean intercepts and slopes associated with each class are shown, and the intercept and slope variances, are equal across classes. Confidence intervals are shown in brackets.

* 95% confidence intervals do not span 1.

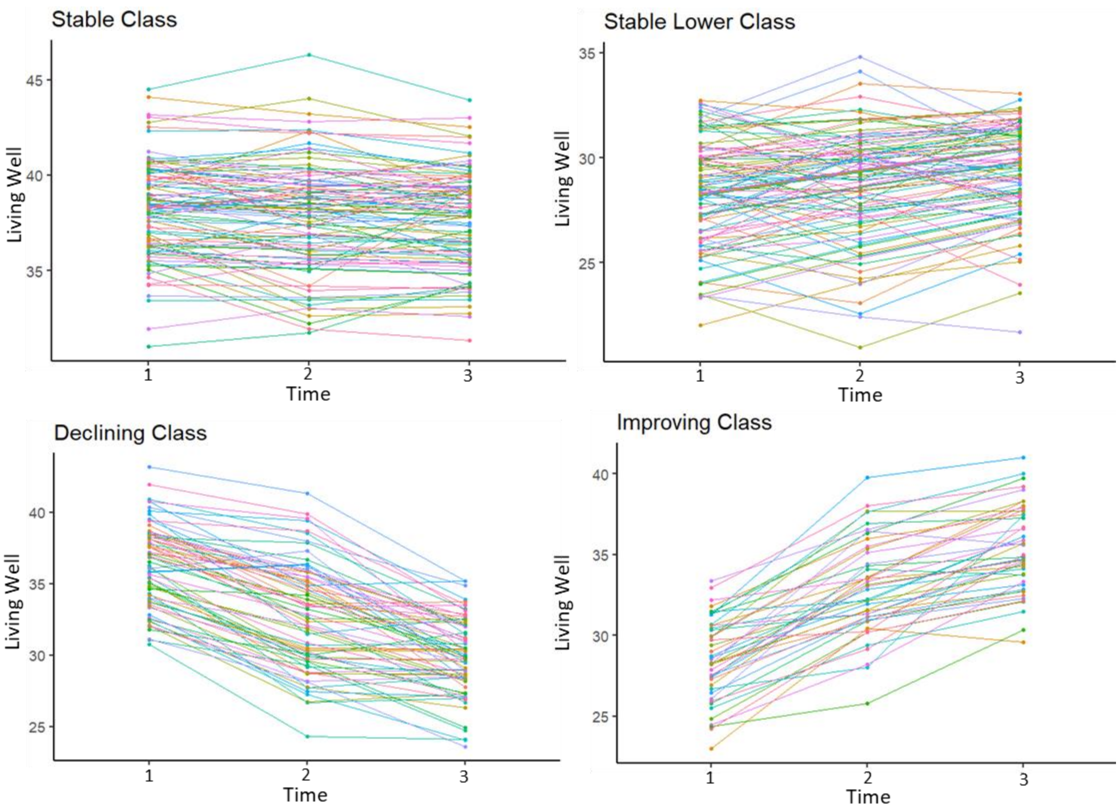

Supplementary Figure S7. Plots of individuals within the four classes of trajectories of 'living well'. The most likely class is allocated based on posterior probabilities for graphing purposes. For the stable lower and stable classes, random samples of 200 people were selected.

Supplementary References

Asparouhov, T., & Muthen, B. (2010). *Multiple Imputation with Mplus*.

Asparouhov, T., & Muthen, B. (2013). *Auxiliary variables in mixture modeling: a 3-step approach using Mplus. Mplus Web Notes: No. 15. Version 6.* .

Brown , T. A. (2006). *Confirmatory factor analysis for applied research*. Guildford Press.

Byrne, B. M., & Watkins, D. (2003). The issue of measurement invariance revisited. *Journal of Cross-Cultural Psychology*, *34*(2), 155-175. https://doi.org/10.1177/0022022102250225

Chen, F. F. (2007). Sensitivity of goodness of fit indexes to lack of measurement invariance. *Structural Equation Modeling*, *14*(3), 464-504. https://doi.org/10.1080/10705510701301834

Cheung, G. W., & Rensvold, R. B. (2002). Evaluating goodness-of-fit indexes for testing measurement invariance. *Structural Equation Modeling*, *9*(2), 233-255. https://doi.org/10.1207/S15328007SEM0902_5

Clare, L., Martyr, A., Henderson, C., Gamble, L., Matthews, F. E., Quinn, C., Nelis, S. M., Rusted, J., Thom, J., Knapp, M., Hart, N., & Victor, C. (2020). Living alone with mild-to-moderate dementia: findings from the IDEAL cohort. *Journal of Alzheimer's Disease*, *78*(3), 1207-1216. https://doi.org/10.3233/JAD-200638

Diggle, P. D., & Kenward, M. G. (1994). Informative dropout in longitudinal data analysis (with discussion). *Applied Statistics*, *43*, 49-73. https://doi.org/10.2307/2986113

Enders, C. K., & Bandalos, D. L. (2001). The relative performance of full information maximum likelihood estimation for missing data in structural equation models. *Structural Equation Modeling*, *8*, 430-457. https://doi.org/10.1207/S15328007SEM0803_5

Grimm, K. J., & Ram, N. (2009). Non-linear growth models in Mplus and SAS. *Structural Equation Modeling*, *16*(4), 676-701. https://doi.org/10.1080/10705510903206055

Hipp, J. R., & Bauer, D. J. (2006). Local solutions in the estimation of growth mixture models. *Psychological Methods*, *11*(1), 36-53. https://doi.org/10.1037/1082-989X.11.1.36

Hu, L., & Bentler, P. M. (1999). Cutoff criteria for fit indexes in covariance structure analysis: Conventional criteria versus new alternatives. *Structural Equation Modeling*, *6*(1), 1-55. https://doi.org/10.1080/10705519909540118

Jung, T., & Wickrama, K. A. S. (2008). An introduction to latent class growth analysis and growth mixture modeling. *Social and Personality Psychology Compass*, *2*(1), 302-317. https://doi.org/10.1111/j.1751-9004.2007.00054.x

Kline, R. B. (2011). *Methodology in the Social Sciences.Principles and practice of structural equation modeling* (3rd ed.). Guildford Press.

Little, T. D. (2013). *Longitudinal structural equation modelling*. Guildford Press.

Millsap, R. E., & Cham, H. (2012). *Investigating factorial invariance in longitudinal data* (B. Laursen, T. D. Little, & N. A. Card, Eds.). The Guilford Press.

Millsap, R. E., & Olivera-Aguilar, M. (2012). *Investigating measurement invariance using confirmatory factor analysis. In R.* The Guilford Press.

Muthen, B. (2004). *Latent variable analysis: growth mixture modeling and related techniques for longitudinal data.* . Sage Publications.

Muthen, B., Asparouhov, T., Hunter, A. M., & Leuchter, A. F. (2011). Growth modeling with nonignorable dropout: alternative analyses of the STAR*D antidepressant trial. *Psychological Methods*, *16*(1), 17-33. https://doi.org/10.1037/a0022634

Muthen, B., & Shedden, K. (1999). Finite mixture modeling with mixture outcomes using the EM algorithm. *Biometrics, 55*, 463-469. https://doi.org/10.1111/j.0006-341X.1999.00463.x

Muthén, L. K., & Muthén, B. O. (1998-2017). *Mplus User's Guide* (8th ed.). Muthén & Muthén.

Nylund, K. L., Asparouhov, T., & Muthen, B. (2007). Deciding on the number of classes in latent class analysis and growth mixture modeling: a Monte Carlo simulation study. *Structural Equation Modeling*, *14*(4), 535-569. https://doi.org/10.1080/10705510701575396

Office for National Statistics. (2010). *Standard Occupational Classification 2010. Volume 3. The National Statistics Socio-economic Classification: (Rebased on the SOC2010) User Manual*. Palgrave Macmillan.

Rouanet, A., Helmer, C., Dartigues, J. F., & Jacqmin-Gadda, H. (2019). Interpretation of mixed models and marginal models with cohort attrition due to death and drop-out. *Statistical Methods in Medical Research*, *28*(2), 343-356. https://doi.org/10.1177/0962280217723675

Rubin, D. B. (1996). Multiple imputation after 18+ years. *Journal of the American Statistical Association*, *91*(434), 473-489. https://doi.org/10.1080/01621459.1996.10476908

Schermelleh-Engel, K., Moosbrugger, H., & Müller, H. (2003). Evaluating the fit of structural equation models: tests of significance and descriptive goodness-of-fit measures. *Methods of Psychological Research*, *8*(2), 23-74.

Tein, J. Y., Coxe, S., & Cham, H. (2013). Statistical power to detect the correct number of classes in latent profile analysis. *Structural Equation Modeling*, *20*(4), 640-657. https://doi.org/10.1080/10705511.2013.824781

Vermunt, J. K. (2010). Latent class modeling with covariates: two improved three-step approaches. *Political Analysis*, *18*, 450-469. https://doi.org/10.1093/pan/mpq025

Wickrama, K. A. S., Lee, T. K., O'Neal, C. W., & Lorenz, F. O. (2016). *Higher-order growth curves and mixture modeling with Mplus. A practical Guide*. Routledge.
